# Characterizing visual read tau‐PET‐negative participants with Alzheimer's disease dementia

**DOI:** 10.1002/alz.14423

**Published:** 2025-04-12

**Authors:** Roos M. Rikken, Emma M. Coomans, Lotte A. de Koning, Denise Visser, Eline Neutelings, Anouk den Braber, Lyduine E. Collij, Sandeep S. V. Golla, for the Alzheimer's Disease Neuroimaging Initiative, Frederik Barkhof, Pieter Jelle Visser, Philip Scheltens, Wiesje M. van der Flier, Ronald Boellaard, Rik Ossenkoppele, Everard G. B. Vijverberg, Elsmarieke van de Giessen, for ADNI

**Affiliations:** ^1^ Radiology & Nuclear Medicine Vrije Universiteit Amsterdam, Amsterdam UMC location VUmc Amsterdam The Netherlands; ^2^ Amsterdam Neuroscience, Brain Imaging, Amsterdam UMC location VUmc Amsterdam The Netherlands; ^3^ Alzheimer Center Amsterdam, Neurology Vrije Universiteit Amsterdam, Amsterdam UMC location VUmc Amsterdam The Netherlands; ^4^ Amsterdam Neuroscience, Neurodegeneration Amsterdam UMC location VUmc Amsterdam The Netherlands; ^5^ Biological Psychiatry Vrije Universiteit Amsterdam Amsterdam The Netherlands; ^6^ Clinical Memory Research Unit, Clinical Sciences Malmö, Faculty of Medicine Lund University, Skånes Universitetssjukhus, VE Minnessjukdomar Malmö Sweden; ^7^ Queen Square Institute of Neurology and Centre for Medical Image Computing University College London London UK; ^8^ Alzheimer Center Limburg, School for Mental Health and Neuroscience Maastricht University Maastricht The Netherlands; ^9^ Department of Neurobiology, Care Sciences and Society, Division of Neurogeriatrics Karolinska Institutet Solna Sweden; ^10^ Epidemiology & Data Science Vrije Universiteit Amsterdam, Amsterdam UMC location VUmc Amsterdam The Netherlands

**Keywords:** Alzheimer's disease dementia, [^18^F]flortaucipir, tau‐PET, visual read

## Abstract

**INTRODUCTION:**

A subset of amyloid beta (Aβ)‐positive Alzheimer's disease (AD) patients is tau‐positron emission tomography (PET) negative. We aimed to characterize this subgroup using [^18^F]flortaucipir PET visual read (VR), as this is important for prognosis and selection for therapies.

**METHODS:**

Aβ‐positive VR tau‐PET‐negative AD dementia patients (AD A+T−) were compared to tau‐PET‐positive AD patients (AD A+T+) and control groups (CU A−T−; CU A+T−) included from the Amsterdam‐based cohort and Alzheimer's Disease Neuroimaging Initiative (ADNI). We compared [^18^F]flortaucipir binding in an early‐ and late‐stage tau ROI, atrophy, cognition, and co‐pathologies.

**RESULTS:**

AD A+T− were older, showed less hippocampal atrophy and slower cognitive decline compared to AD A+T+. In ADNI, AD A+T− showed higher early‐stage tau binding compared to both control groups and more late‐stage tau compared to CU A−T−, but no tau accumulation over time.

**DISCUSSION:**

VR tau‐PET‐negative AD patients show neurodegenerative and cognitive processes consistent with the AD trajectory, but milder progression compared to tau‐PET‐positive AD patients.

**Highlights:**

- We used the novel Food and Drug Administration (FDA)‐approved VR method for defining tau‐PET positivity.
- AD A+T− patients were older and showed less atrophy and cognitive decline than AD A+T+.
- We did not find convincing evidence of tau accumulation in AD A+T− or copathologies.
- The group of AD A+T− patients is likely very heterogeneous.

## 1 BACKGROUND

The accumulation of amyloid‐beta (Aβ) plaques and neurofibrillary tau tangles are neuropathological hallmarks of Alzheimer's disease (AD). Aβ biomarkers are widely implemented in clinical decision‐making and can support an AD diagnosis.^1^ Tau biomarkers are also increasingly being implemented in clinical practice and show promise for both diagnostic and prognostic purposes, as tau biomarkers demonstrate a higher specificity for AD and are more closely associated with cognitive decline and disease progression.^2^, ^3^, ^4^, ^5^, ^6^, ^7^, ^8^, ^9^

Neurofibrillary tau tangles can be visualized and quantified in vivo using positron emission tomography (PET). Recently, a method for the visual assessment of [^18^F]flortaucipir (tau‐)PET scans was approved for clinical use by the US Food and Drug Administration (FDA) to support the diagnosis of AD dementia. This visual read (VR) method shows high accuracy for detecting late‐stage tau pathology, as a positive scan is strongly suggestive of the presence of neuropathological tau in Braak stages V‐VI.^10^, ^11^ Tau‐tracer binding in early‐stage tau regions (i.e. the medial temporal lobe [MTL]) does not contribute to a tau‐positive VR. We and others previously found that approximately 10% to 25% of Aβ‐positive individuals with AD dementia have a negative tau‐PET VR.^12^, ^13^, ^14^ Accordingly, individuals could have increased tau‐PET tracer binding in medial temporal areas, but a negative tau‐PET VR and thereby may escape detection by VR.

Alternatively, the presence of co‐pathologies (in addition to Aβ pathology) could contribute to cognitive impairment in individuals with low levels of tau‐PET binding. For example, previous studies indicated that AD patients with comorbid cerebrovascular disease showed tau pathology in lower Braak stages,^15^ and the presence of Lewy bodies and TAR DNA‐binding protein 43 (TDP‐43) co‐pathology has been associated with faster cognitive decline in AD.^16^, ^17^, ^18^ Accordingly, the National Institute on Aging‐Alzheimer's Association (NIA‐AA) guidelines for neuropathological assessment propose that in cognitively impaired individuals with higher Aβ levels and lower Braak stages, the relative contribution of vascular and alpha‐synuclein co‐pathologies should be considered, highlighting the importance of examining these co‐pathologies in Aβ‐positive individuals with dementia with lower levels of tau‐PET binding.^19^ This is also reflected in the revised AA's criteria for diagnosis and staging of AD which incorporates biomarkers of non‐AD co‐pathology, including vascular and alpha‐synuclein pathologies.^20^

Currently, Aβ‐positive symptomatic individuals with clinical AD dementia and a negative tau‐PET VR are not yet well characterized, and their longitudinal disease trajectory is unclear. A recent study characterized a group of Aβ‐positive cognitively impaired individuals (both mild cognitive impairment [MCI] and dementia) who were tau‐PET‐negative based on a global quantitative threshold and were found to be older and more often male and showed less cognitive impairment.^21^ Here, we focussed on Aβ‐positive participants with a clinical diagnosis of AD dementia (as individuals with MCI may be on their way to developing tau pathology and reflect earlier stages of disease progression) and defined tau positivity based on VR, which is currently the only FDA‐approved method for defining the presence of tau in the clinic and is pathologically verified.^10^, ^12^ Hence, the overarching aim was to obtain a detailed characterization of individuals with a clinical diagnosis of AD dementia who were Aβ‐positive but VR tau‐PET‐negative. To investigate whether tau‐negative participants with an AD dementia diagnosis were on a trajectory toward developing tau, we studied baseline and longitudinal quantitative tau uptake. We additionally investigated whether VR tau‐negative participants with AD dementia had differential atrophy patterns and cognitive trajectories compared to tau‐positive participants with AD dementia and control groups. Lastly, we investigated whether tau‐negative participants with AD dementia had greater vascular or Lewy body burden.

## 2 METHODS

### 2.1 Participants

Participants were included from two cohorts. The Amsterdam‐based cohort included participants who underwent [^18^F]flortaucipir PET for research purposes at Amsterdam University Medical Center (AUMC) and largely consisted of participants from the Amsterdam Dementia Cohort,^22^ the Subjective Cognitive Impairment Cohort,^23^ and the EMIF‐AD PreclinAD study (for further information see ref. ^12^). The open‐access Alzheimer's Disease Neuroimaging Initiative (ADNI) cohort was added as another independent cohort. The ADNI was launched in 2003 as a public‐private partnership, led by principal investigator Michael W. Weiner, MD. The primary goal of ADNI has been to test whether serial magnetic resonance imaging (MRI), PET, and clinical and neuropsychological assessment can be combined to measure the progression of MCI and early AD. For up‐to‐date information, see https://adni.loni.usc.edu/. For both the Amsterdam‐based cohort and ADNI, participants were included if they had Aβ status, tau‐PET data, and a clinical diagnosis available.

Our primary participant group of interest included Aβ‐positive patients with a clinical diagnosis of probable AD dementia who were visually read as tau‐PET negative (AD A+T−). We compared this group to Aβ‐positive AD dementia patients who were visually read as tau‐PET positive (AD A+T+) (details on amyloid and tau positivity described below). We additionally included two control groups: one cognitively unimpaired group with an identical biomarker profile as our primary participant group of interest (CU A+T−) and one cognitively unimpaired group negative on both amyloid and tau (CU A−T−) as a true negative control group. Additionally, we included MCI as a separate group solely in ADNI, due to the small sample size of MCI A+T− in the Amsterdam‐based cohort (*N* = 2). As patients with a MCI diagnosis are still at an earlier disease stage than participants with dementia and the cause of tau negativity may differ from tau‐PET‐negative AD dementia patients, we analyzed the MCI group separately as an additional group of interest.

**RESEARCH IN CONTEXT**

1. **Systematic review**: The authors reviewed relevant literature using PubMed. Several studies focus on tau‐PET positivity across dementias using a quantitative cut‐off. Only recently was the VR tau‐PET positivity investigated in AD and DLB. However, less is known about VR tau‐PET‐negative AD dementia patients.
2. **Interpretation**: Our findings indicate that amyloid‐positive VR tau‐PET‐negative AD patients are older and have less cognitive decline compared to VR tau‐PET‐positive AD patients but do show more cognitive decline and atrophy compared to controls, indicating underlying neurodegenerative processes with milder disease progression.
3. **Future directions**: This study characterized tau‐PET‐negative AD patients in detail. These findings are important for the use of tau‐PET in clinical practice, patient prognosis, and selection for therapies. Studies with a larger sample with longer tau‐PET follow‐up are necessary to further characterize these patients.

### 2.2 Amyloid‐PET and CSF p‐tau 181

In the Amsterdam‐based cohort, Aβ status was determined by either CSF (*N* = 62) or PET (*N* = 154) closest to the tau‐PET date. Aβ‐PET status was determined by [^18^F]florbetapir, [^18^F]flutemetamol, [^18^F]florbetaben PET VR according to company guidelines, or [^11^C] Pittsburgh compound B (PiB) according to previously published methods.^24^ In ADNI, Aβ positivity was determined by [^18^F]florbetapir, [^18^F]florbetaben, or [^11^C]PiB PET using previously published cut‐offs (≥ 1.11 standardized uptake value ratio [SUVR] [^18^F]florbetapir; ≥ 1.08 [^18^F]florbetaben, ≥ 1.465 [^11^C]PiB PET).^25^, ^26^ Additionally, Centiloids were compared between the groups in ADNI. CSF p‐tau 181 was available for a subset of participants (CSF p‐tau availability Amsterdam‐based cohort: CU A−T−: 10/92, CU A+T− 6/39, AD A+T− 4/10, AD A+T+ 34/75. CSF p‐tau availability ADNI: CU A−T−: 148/268, CU A+T− 60/112, AD A+T− 11/18, AD A+T+ 30/58). In the Amsterdam‐based cohort, the Innotest CSF p‐tau 181 was used to measure p‐tau. In ADNI, the Roche Elecsys assay was used.

### 2.3 Tau‐PET acquisition, VR, and quantification

In the Amsterdam‐based cohort, all participants underwent a dynamic [^18^F]flortaucipir PET scan acquired on a Philips Ingenuity TF‐64 PET/CT scanner. A subset of participants (*N* = 80, 37%) had a follow‐up scan available acquired 0.9 to 4.7 years (mean 2.06 ± 0.51, maximum two visits) after baseline tau‐PET. Low‐dose CT scans were acquired prior to the start of the PET scan to account for attenuation correction. Several dynamic acquisition protocols were used; all acquisitions included at least 0 to 30 and 80 to 100 min after injection, as described elsewhere.^27^, ^28^ Receptor parametric mapping (RPM) was used to obtain parametric images of BP_ND_ with the cerebellar gray matter as a reference region. In ADNI, [^18^F]flortaucipir PET images were acquired using 3D acquisitions of six 5‐min frames 75 min after injection. Again, a subset of participants (*N* = 220, 48.2%) underwent longitudinal tau‐PET follow‐up (mean: 1.04 ± 1.5 years, maximum: 5.9 years, maximum four visits). For ADNI, SUVRs were calculated with the cerebellar gray matter as a reference region. Details on data acquisition and analysis can be found on the ADNI site.^29^ While in ADNI we only had SUVR available, we included BP_ND_ in the Amsterdam‐based cohort as BP_ND_ is less biased toward changes in blood flow, which is especially important when assessing longitudinal changes in tau‐PET binding.^30^

Tau‐PET VR status was defined by [^18^F]flortaucipir PET VR according to US FDA‐approved guidelines on summed 80‐ to 100‐min post‐injection frames.^10^ In Amsterdam, [^18^F]flortaucipir PET VR was performed by two nuclear medicine physicians blinded to clinical information, as described previously.^12^ Discordant reads were re‐evaluated in a joint consensus meeting, which resulted in a consensus VR. For ADNI, tau‐PET VR was performed for the current study by a trained reader in Amsterdam. If there was any doubt about the result of the read, a second reader evaluated the scans. Again, discordant scans were re‐evaluated, resulting in a consensus VR. Only increased signal in posterolateral temporal, occipital, and/or parietal/precuneus region(s) in either hemisphere resulted in a positive VR. No increased signal or increased signal outside of the aforementioned regions (including medial temporal) resulted in a negative VR.

We quantified [^18^F]flortaucipir binding in two regions of interest (ROI): (1) in an early‐stage tau ROI (ie, the MTL) that is not included in a positive VR and (2) a late‐stage tau ROI composed of all regions included in a positive VR according to FDA guidelines (ie, posterolateral temporal, parietal and occipital lobe). In the Amsterdam‐based cohort, the MTL was composed of the entorhinal cortex from the Svarer atlas and the amygdala, fusiform gyrus, and parahippocampal gyri from the Hammers atlas.^31^, ^32^ In ADNI, the MTL was composed of the entorhinal cortex, amygdala, fusiform gyrus, and parahippocampal gyri based on the FreeSurfer atlas. The late‐stage region was composed of posterolateral temporal, parietal, and occipital regions from the Hammers atlas in the Amsterdam‐based cohort and the FreeSurfer atlas in ADNI.

### 2.4 MRI analyses: hippocampal volume, cortical thickness, and vascular pathology

Cortical thickness (mm) measures were obtained using FreeSurfer. In the Amsterdam‐based cohort and ADNI, participants underwent MRI scanning on a 3T MRI scanner. If multiple MRI scans were available, the MRI closest to tau‐PET was used. In Amsterdam, T1 and Fluid Attenuated Inversion Recovery (FLAIR) images were used as input to achieve optimal segmentation. If FLAIR was not available, only T1 was used as input (see ref. [^30^]). In ADNI, only T1 images were used as input. We included two measures of brain atrophy: hippocampal volume and global cortical thickness. Hippocampal volume was corrected for intracrianial volume (ICV). Global cortical thickness was based on an AD‐signature ROI including the following regions: entorhinal cortex, inferior temporal, temporal pole, angular gyrus, superior frontal, superior parietal, supramarginal, precuneus, and inferior frontal.^33^

For vascular pathology, we determined the extent of white matter hyperintensities (WMHs) lesions and the presence of infarcts. In Amsterdam, WMH lesions were quantified on T1 and FLAIR using a lesion growth algorithm as implemented in the lesion segmentation tool (LST) for SPM12.^34^ Lacunar infarcts were visually rated by a radiologist or trained reader as part of the routine clinical workup. In ADNI, WMH lesions were quantified using an automatic WMH segmentation pipeline using T1 and FLAIR sequences processed at the University of California at Davis. MRI scans were reviewed for the detection of lacunar and cortical infarcts by a trained physician. For the visualization of concomitant co‐pathology, WMH positivity (WMH+) was defined by >1.5 SD with reference to the subset of <65‐year CU A−T−.

2.5

To detect alpha‐synuclein co‐pathology, the presence of alpha‐synuclein seeds in the CSF was assessed by the Amprion synuclein seed amplification assay based on available data in ADNI (not available in the Amsterdam‐based cohort).^35^, ^36^ The Amprion synuclein seed amplification assay detects misfolded α‐synuclein aggregate seeds, resulting in a detected (positive), intermediate, or undetected (negative) signal. Intermediate results were removed. Detected alpha‐synuclein aggregate seeds can be classified as Type 1, as seen in Parkinson's disease or Lewy body dementia, or Type 2, consistent with multiple system atrophy. Only a subset of participants had Amprion synuclein seed amplification assay data available: CU A−T− *N* = 197/268 [74%], CU A+T− 82/112 [73%], AD A+T− 12/18 [75%], AD A+T+ 44/58 [75%].

### 2.6 Cognition

For both the Amsterdam‐based cohort and ADNI we included cognitive tests covering both memory and non‐memory domains. For the Amsterdam‐based cohort, memory domain and non‐memory domain scores were created. The memory domain score consisted of the RAVLT immediate and delayed recall. The non‐memory domain score consisted of the trail‐making test (parts A and B) and animal fluency. All scores were standardized to the mean and standard deviation of a larger cohort of 440 cognitively unimpaired Aβ‐negative individuals from the ADC to increase accuracy, resulting in an individual *z*‐score for both composite domain scores. Non‐memory time points were excluded if at least two tests were missing in the non‐memory domain. The majority of participants (*N* = 208, 91.2%) had longitudinal data available over a period of 8 years from before and after the time of baseline tau‐PET (range 1 to 10 visits; median three visits). In ADNI, domain scores for memory, executive functioning, and language available in ADNI were used.^37^, ^38^, ^39^ Three hundred seventy‐one participants (81.4%) had longitudinal cognition data available ranging from 15 years before tau‐PET till 6 years after tau‐PET (range 1 to 13 visits; median two visits).

### 2.7 Statistical analysis

Analyses were performed separately per cohort. In all analyses, AD A+T− was used as the reference group and compared to the CU A−T− (true control group), CU A+T− (identical biomarker profile, different cognitive stage), and AD A+T+ (different biomarker profile, identical cognitive stage) groups. Patient characteristics were compared between groups using *t* tests and Fisher's exact tests. Linear mixed models with random intercepts and fixed slopes with an interaction of group × time corrected for age and sex were used to compare regional baseline and longitudinal tau binding between groups, which were statistically considered to be the best fit (tau binding ~ group × time between visits + group + time + sex + age + (1|Subject)). Linear regressions corrected for age and sex were used to detect differences in hippocampal volume and global cortical thickness. Linear regressions corrected for age, sex, and Apolipoprotein E (*APOE)* ε4 carriership were used to compare WMH volume between groups.^40^, ^41^ The difference between groups in the presence of infarcts and alpha‐synuclein aggregate seeds was compared using Fisher's exact tests. Baseline and longitudinal cognition (including Mini‐Mental State Examination [MMSE]) were assessed with linear mixed models with random intercept and fixed slope with an interaction of group × time with age, sex, and education as covariates (cognitive domain or MMSE ~ group × time between visits + group + time + sex + age + education + (1|Subject)). Linear regressions corrected for age were used to detect differences in Centiloids between the groups. R version 4.2.1 was used for statistical analyses and visualization of data. *P*‐values <0.05 were considered to be significant.

## 3 RESULTS

### 3.1 Participant characteristics

Participant characteristics are shown in Table 1. In the Amsterdam‐based cohort and ADNI, 12% (*N* = 18) and 24% (*N* = 10) of the amyloid‐positive AD dementia group were VR tau‐PET negative respectively. AD A+T− were significantly older than AD A+T+ in both Amsterdam and ADNI (Amsterdam: 74.1 ± 4.8 vs. 64.6 ± 7.5, *p* < 0.001; ADNI 81.8 ± 6.6 vs. 76.3 ± 9.3, *p* = 0.009). In the Amsterdam‐based cohort, AD A+T− participants were more often male compared to AD A+T+ participants (*p* = 0.002), but this was not the case in ADNI (*p* = 0.418). In both cohorts, MMSE at baseline was higher in AD A+T− compared to AD A+T+ (Amsterdam: *β* = −0.86, *p* < 0.001; ADNI: *β* = −1.03, *p* < 0.001), and, AD A+T− tended to be more often *APOE* ε4 carrier compared to AD A+T+ in ADNI, although at trend level (ADNI: *p* = 0.05). No significant differences in years of education (Amsterdam: *p* = 0.779, ADNI: *p* = 0.726) in either cohort or prevalence of *APOE* ε4 carriership in the Amsterdam‐based cohort (*p* = 0.715) were found between AD A+T− and AD A+T+. Group comparisons between AD A+T− and the CU groups can be found in Table 1.

**TABLE 1:** Participant characteristics.

	Amsterdam‐based cohort				ADNI			
	CU A−T− (*N* = 92)	CU A+T− (*N* = 39)	AD A+T− (*N* = 10)	AD A+T+ (*N* = 75)	CU A−T− (*N* = 268)	CU A+T− (*N* = 112)	AD A+T− (*N* = 18)	AD A+T+ (*N* = 58)
Age	69.6 (7.1)^a^	71.5 (9.1)	74.1 (4.8)	64.6 (7.5)^c^	71.4 (7.4)^a^	74.9 (7.9)^b^	81.8 (6.6)	76.3 (9.3)^c^
Sex, M (%)	48 (52.8)^a^	20 (51.3)^b^	10 (100)	39 (52)^c^	111 (41.4)^a^	44 (39.3)^b^	12 (66.7)	31 (53.4)
Education in years	12.4 (2.9)	12.4 (2.8)	12.3 (2.5)	12.1 (3.0)	16.8 (2.3)	16.7 (2.3)	15.6 (2.9)	15.3 (2.3)
APOE ε4, *n* carrier (%)	27 (31.4)^a^	25 (65.8)	8 (80)	49 (69)	56 (23.7)	51 (51)	7 (46.7)	41 (75.9)
MMSE	29.0 (1.2)^a^	28.7 (1.3)^b^	23.2 (3.4)	21.0 (4.3)^c^	29.1 (1.2)^a^	29.1 (1.1)^b^	24.5 (3.7)	21.8 (1.2)^c^
CSF p‐tau 181^*^	48.7 (26.7)	64.7 (30.1)	74.3 (15.7)	91.7 (36.0)	18.7 (6.8)^a^	25.4 (12.5)	27.1 (9.5)	36.7 (12.3)^c^
Early‐stage tau ROI binding^*^	−0.01 (0.06)	0.06 (0.11)	0.06 (0.11)	0.42 (0.18)^c^	1.15 (0.08)^a^	1.19 (0.09)^b^	1.28 (0.11)	1.77 (0.40)^c^
Late‐stage tau ROI binding^*^	0.06 (0.05)	0.07 (0.05)	0.06 (0.08)	0.64 (0.39)^c^	1.51 (0.09)^a^	1.54 (0.10)	1.57 (0.11)	2.20 (0.61)^c^
Global cortical thickness (mm)	2.67 (0.17)	2.64 (0.19)	2.55 (0.12)	2.60 (0.17)	2.72 (0.12)^a^	2.70 (0.15)^b^	2.52 (0.14)	2.40 (0.20)^c^
Hippocampal volume (mL)	3831.39 (485.49)^a^	3656.51 (469.69)^b^	3422.34 (524.80)	3170.87 (452.11)^c^	3821.44 (437.12)^a^	3736.88 (381.21)^b^	2963.95 (567.28)	2879.01 (454.11)^c^
WMH (log)	0.55 (1.59)^a^	0.33 (1.54)	1.63 (1.30)	1.29 (1.29)	0.09 (1.41)	0.80 (1.39)	0.99 (1.53)	1.62 (1.03)^c^
Infarct, *n* (%)	0 (0)	3 (20)	2 (22.2)	8 (11.4)	25 (11.1)	13 (13)	3 (21.4)	7 (15.6)
Alpha‐synuclein, *n* (%)					30 (15.2)^a^	15 (18.3)	5 (41.7)	18 (40.9)

*Note*: Age, education, and MMSE are shown as mean (SD).

Abbreviations: AD, Alzheimer's disease; CU, cognitively unimpaired; MMSE, Mini‐Mental State Examination; WMH, white matter hyperintensity.

^a^ Significantly different between AD A+T− and CU A−T−.

^b^ Significantly different between AD A+T− and CU A+T−.

^c^ Significantly different between AD A+T− and AD A+T+.

^*^ CSF p‐tau 181 results were not compared in Amsterdam‐based cohort due to low availability. Binding represents BP_ND_ in the Amsterdam‐based cohort and SUVR in ADNI. CSF p‐tau availability Amsterdam‐based cohort: CU A−T−: 10/92, CU A+T− 6/39, AD A+T− 4/10, AD A+T+ 34/75. CSF p‐tau availability ADNI: CU A−T−: 148/268, CU A+T− 60/112, AD A+T− 11/18, AD A+T+ 30/58. WMH availability Amsterdam‐based cohort: CU A−T−: 91/92, CU A+T− 37/39, AD A+T− 9/10, AD A+T+ 73/75. WMH availability ADNI: CU A−T−: 265/268, CU A+T− 112/112, AD A+T− 17/18, AD A+T+ 57/58. Infarct availability ADNI: CU A−T−: 225/268, CU A+T− 100/112, AD A+T− 14/18, AD A+T+ 45/58. Infarct availability in Amsterdam‐based cohort: CU A−T−: 38/92, CU A+T− 15/39, AD A+T− 10/10, AD A+T+ 75/75. APOE ε4 availability ADNI: CU A−T−: 235/268, CU A+T− 100/112, AD A+T− 15/18, AD A+T+ 54/58. APOE ε4 availability in Amsterdam‐based cohort: CU A−T−: 86/92, CU A+T− 38/39, AD A+T− 10/10, AD A+T+ 71/75. Hippocampal volume availability in ADNI: CU A−T−: 200/268, CU A+T− 89/112, AD A+T− 14/18, AD A+T+ 44/58. Hippocampal volume availability in Amsterdam‐based cohort: CU A−T−: 88/92, CU A+T− 37/39, AD A+T− 10/10, AD A+T+ 72/75. Global cortical thickness availability in ADNI: CU A−T−: 200/268, CU A+T− 82/112, AD A+T− 10/18, AD A+T+ 39/58. Global cortical thickness availability in Amsterdam‐based cohort: CU A−T−: 85/92, CU A+T− 37/39, AD A+T− 10/10, AD A+T+ 72/75. Alpha‐synuclein availability in ADNI: CU A−T−: 197/268, CU A+T− 82/112, AD A+T− 12/18, AD A+T+ 44/58.

### 3.2 Baseline and longitudinal changes in tau binding

**FIGURE 1:**
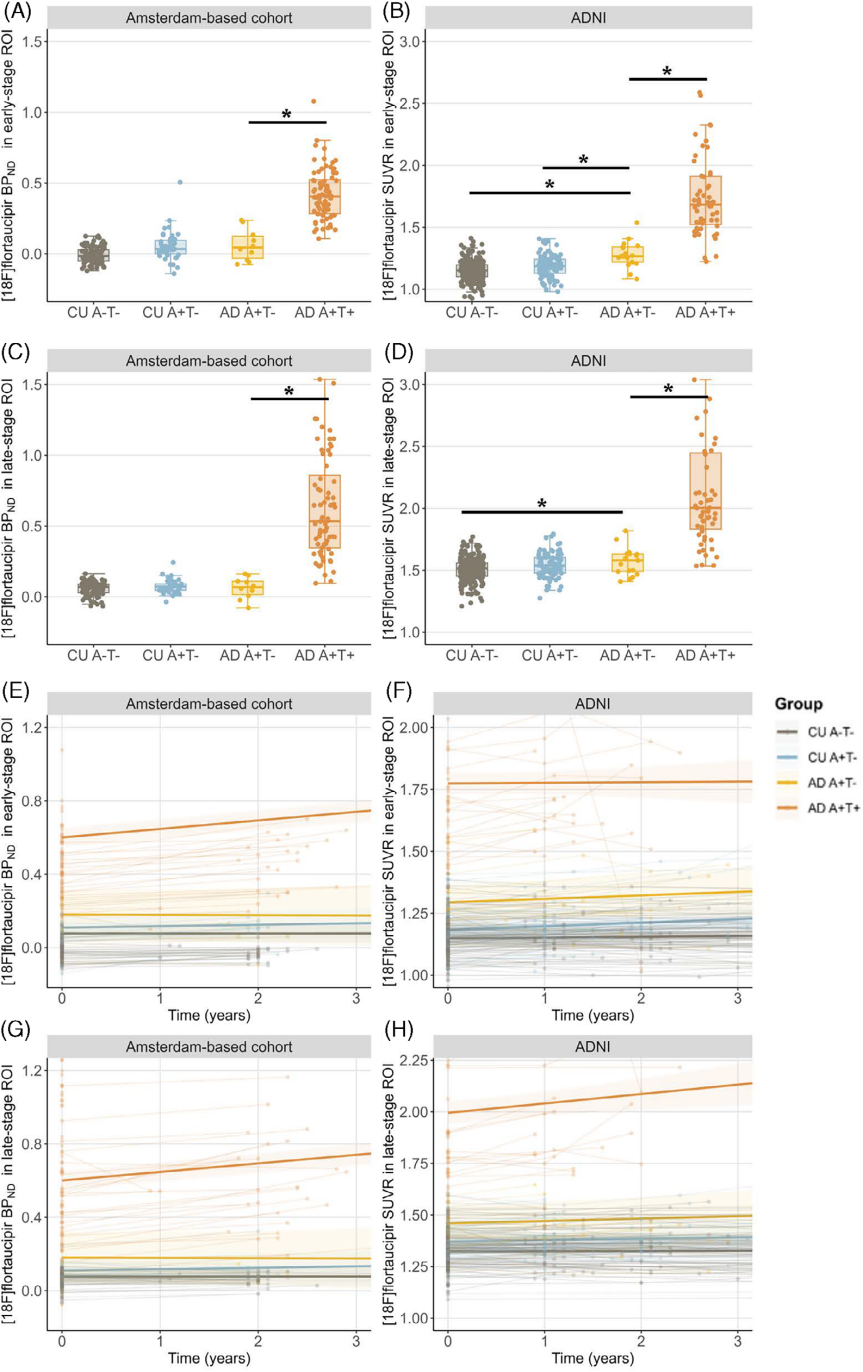
Baseline and longitudinal [^18^F]flortaucipir binding in early‐ and late‐stage tau ROIs. (A–D) Boxplots showing baseline [^18^F]flortaucipir binding in early‐ (MTL) and late‐stage tau ROIs per group. (E–H) Plots showing [^18^F]flortaucipir binding in early‐ and late‐stage tau ROIs per group over time. The reported *p* values are derived from the baseline values of an age‐ and sex‐adjusted linear mixed model. Longitudinal tau‐PET data available in Amsterdam: AD A+T− 3/10; AD A+T+ 27/75; CU A−T− 31/92; CU A+T− 14/39. Logitudinal tau‐PET data available in ADNI: AD A+T−12/18; AD A+T+ 23/58; CU A−T− 114/268; CU A+T− 71/112. MTL, medial temporal lobe; PET, positron emission tomography; ROI, region of interest. * *p* < 0.05.

In ADNI, AD A+T− had a higher baseline tau‐PET SUVR in the early‐stage tau ROI compared to both control groups and a higher baseline tau‐PET SUVR in the late‐stage tau ROI compared to CU A−T− (Figure 1 and Table 1) (all estimates and *p* values are listed in Table S1). There were no significant differences in baseline tau‐PET BP_ND_ in the early‐ or late‐stage tau ROI between AD A+T− and the control groups in the Amsterdam‐based cohort. In both cohorts, AD A+T− also quantitatively showed less tau‐PET binding in the early‐ and late‐stage tau ROIs compared to AD A+T+.

Over time, AD A+T− did not show significantly different tau accumulation trajectories in the early or late tau ROI compared to CU A−T− and CU A+T− in either cohort (Figure 1 and Table S1). Interestingly, the slopes of tau accumulation in the early‐ and late‐stage tau ROIs did not differ between AD A+T− and AD A+T+ in ADNI, indicating similar rates of tau accumulation. By contrast, in the Amsterdam‐based cohort, AD A+T− showed significantly slower rates of tau accumulation compared to AD A+T+ in both ROIs. Similar results were obtained when using SUVR in the Amsterdam‐based cohort (Tables S1 and S2).

### 3.3 Baseline and longitudinal changes in cognition

**FIGURE 2:**
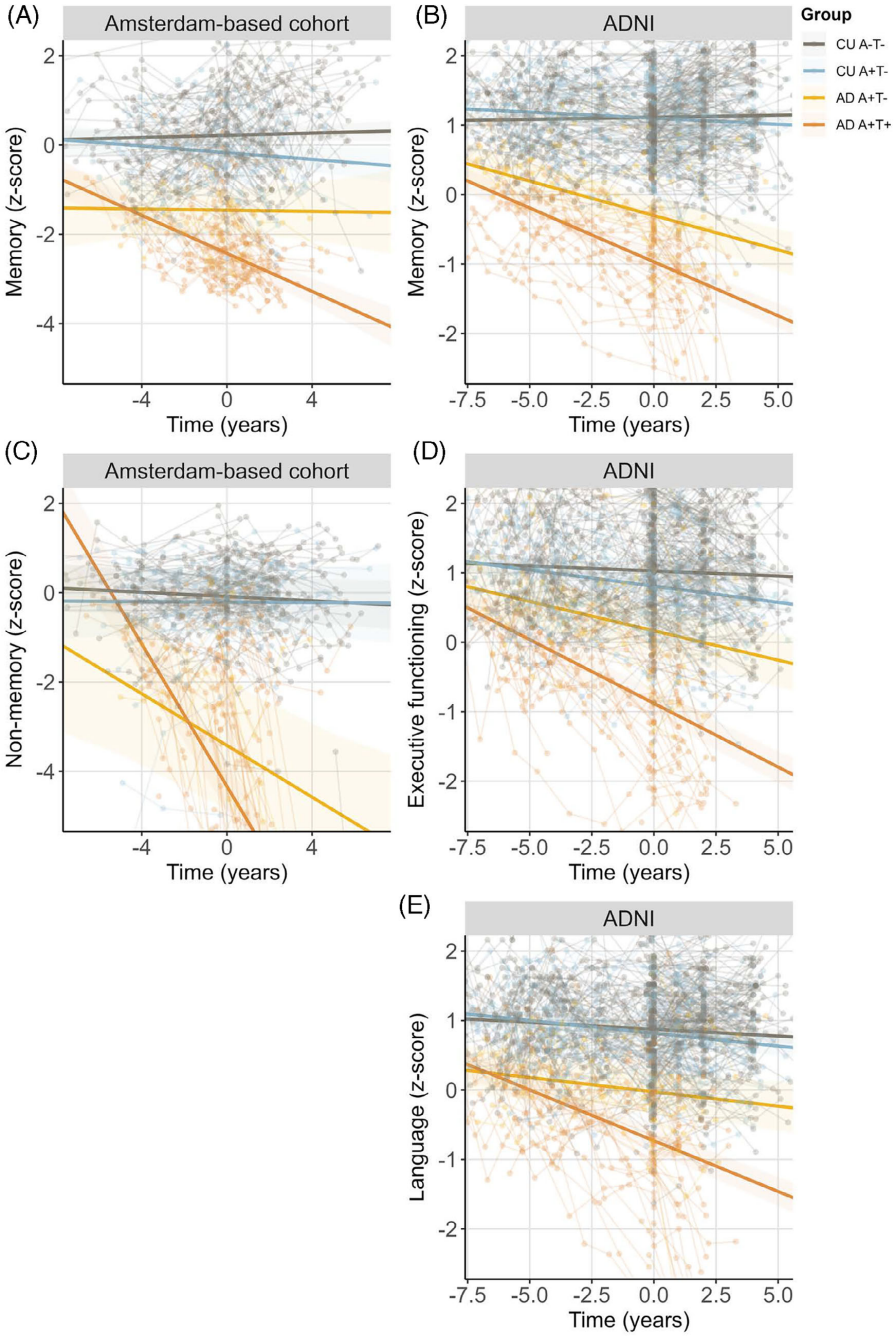
Longitudinal cognition. (A, B) Plot showing composite memory domain *z*‐scores over time in the Amsterdam‐based cohort and ADNI. (C) Plot showing composite non‐memory domain *z*‐scores over time in the Amsterdam‐based cohort. (D) Plot showing composite executive functioning domain *z*‐scores over time in ADNI. (E) Plot showing composite language domain *z*‐scores over time in ADNI. Time is relative to baseline [^18^F]flortaucipir PET, a positive value meaning after [^18^F]flortaucipir PET and a negative value meaning before [^18^F]flortaucipir PET. ADNI, Alzheimer's Disease Neuroimaging Initiative; PET, positron emission tomography.

In ADNI, AD A+T− showed less impairment in memory, executive functioning, and language compared to AD A+T+ at baseline. Likewise, in the Amsterdam‐based cohort, AD A+T− showed less impairment in memory domain scores compared to AD A+T+, but there were no significant differences between AD A+T− and AD A+T− in non‐memory scores at baseline (Figure 2). As expected, AD A+T− showed worse cognitive scores on all domains compared to both control groups in the Amsterdam‐based cohort and ADNI (estimates and *p* values listed in Tables S3 and S4).

Over time, AD A+T− showed a slower decline in memory, executive functioning, and language domains in ADNI and a slower decline in memory and non‐memory compared to AD A+T+ in the Amsterdam‐based cohort (Figure 2, Tables S3 and S4). Nonetheless, AD A+T− showed a steeper decline compared to both control groups in the non‐memory domain in the Amsterdam‐based cohort. In ADNI, AD A+T− showed a steeper decline in memory and executive functioning domains, but not in the language domain (Tables S3 and S4). Similar results were obtained for baseline and longitudinal MMSE scores (Figure S1 and Table S5).

### 3.4 Atrophy

**FIGURE 3:**
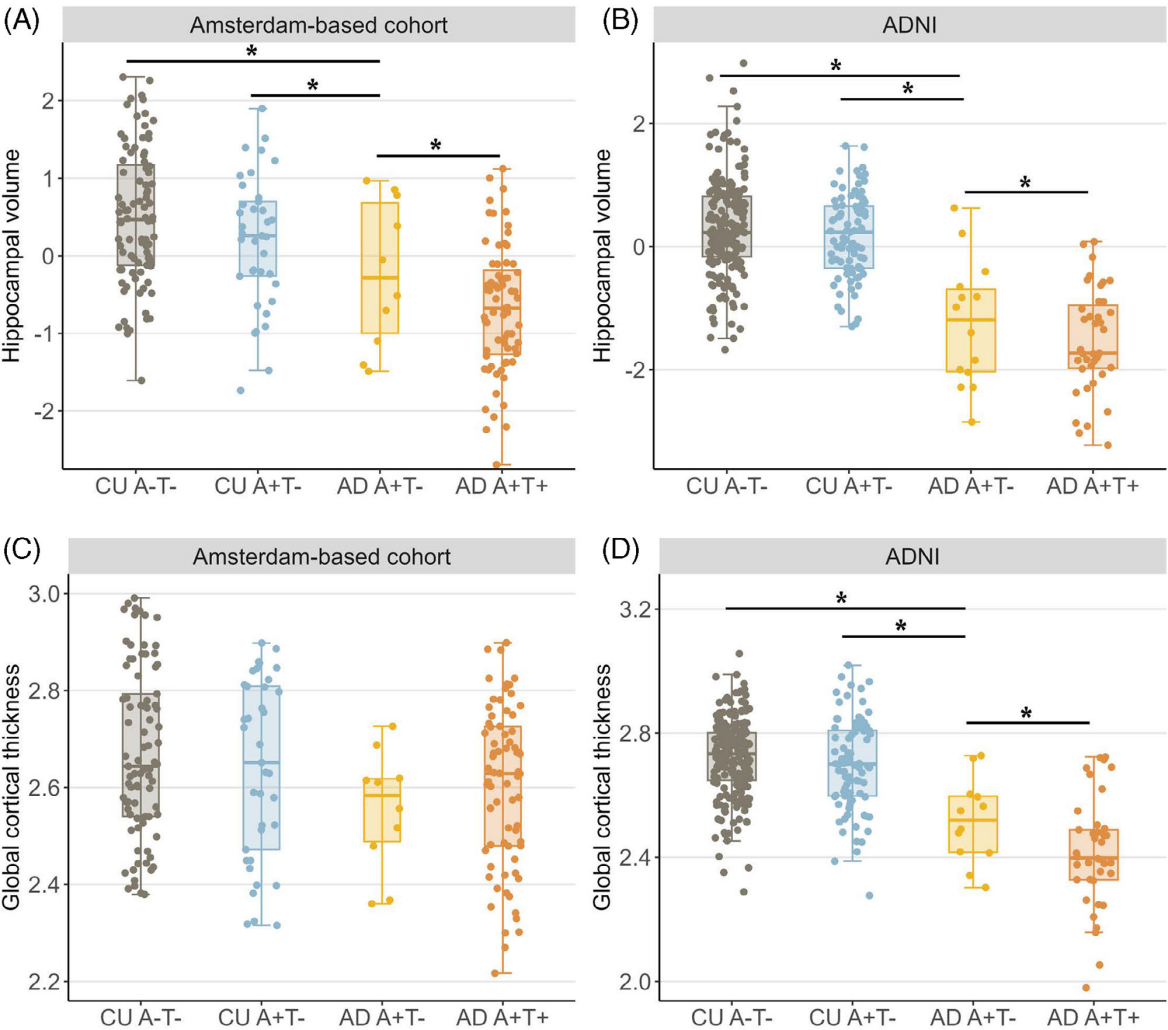
Hippocampal volume and cortical thickness. (A) Boxplot showing residuals of hippocampal volume corrected for intracranial volume in the Amsterdam‐based cohort. (B) Boxplot showing residuals of hippocampal volume corrected for intracranial volume in ADNI. (C) Boxplot showing global cortical thickness in millimeters in the Amsterdam‐based cohort. (D) Boxplot showing global cortical thickness in millimeters in ADNI. Missing ADNI: AD A+T− 6/18 global cortical thickness, 4/17 hippocampal volume; AD A+T+ hippocampal volume 19/58, global cortical thickness 18/58; CU A−T− global cortical thickness 62/268, hippocampal volume 67/268; CU A+T− hippocampal volume 23/112, global cortical thickness 24/112. Missing Amsterdam: AD A+T− global cortical thickness 0/10, hippocampal volume 0/10; AD A+T+ global cortical thickness 3/75 hippocampal volume 3/75; CU A−T− global cortical thickness, 7/92 hippocampal volume 4/92; CU A+T− global cortical thickness 2/39, hippocampal volume 2/39. ADNI, Alzheimer's Disease Neuroimaging Initiative. * *p* < 0.05.

In both cohorts, AD A+T− showed a larger hippocampal volume compared to AD A+T+ but a smaller hippocampal volume compared to both control groups (Table 1, Figure 3, and Table S6). In ADNI, AD A+T− additionally showed higher global cortical thickness compared to AD A+T+ but a lower global cortical thickness compared to both control groups. In the Amsterdam‐based cohort, AD A+T− did not show significant differences in global cortical thickness compared to all other groups.

### 3.5 Co‐pathology: Vascular and alpha‐synuclein contribution

**FIGURE 4:**
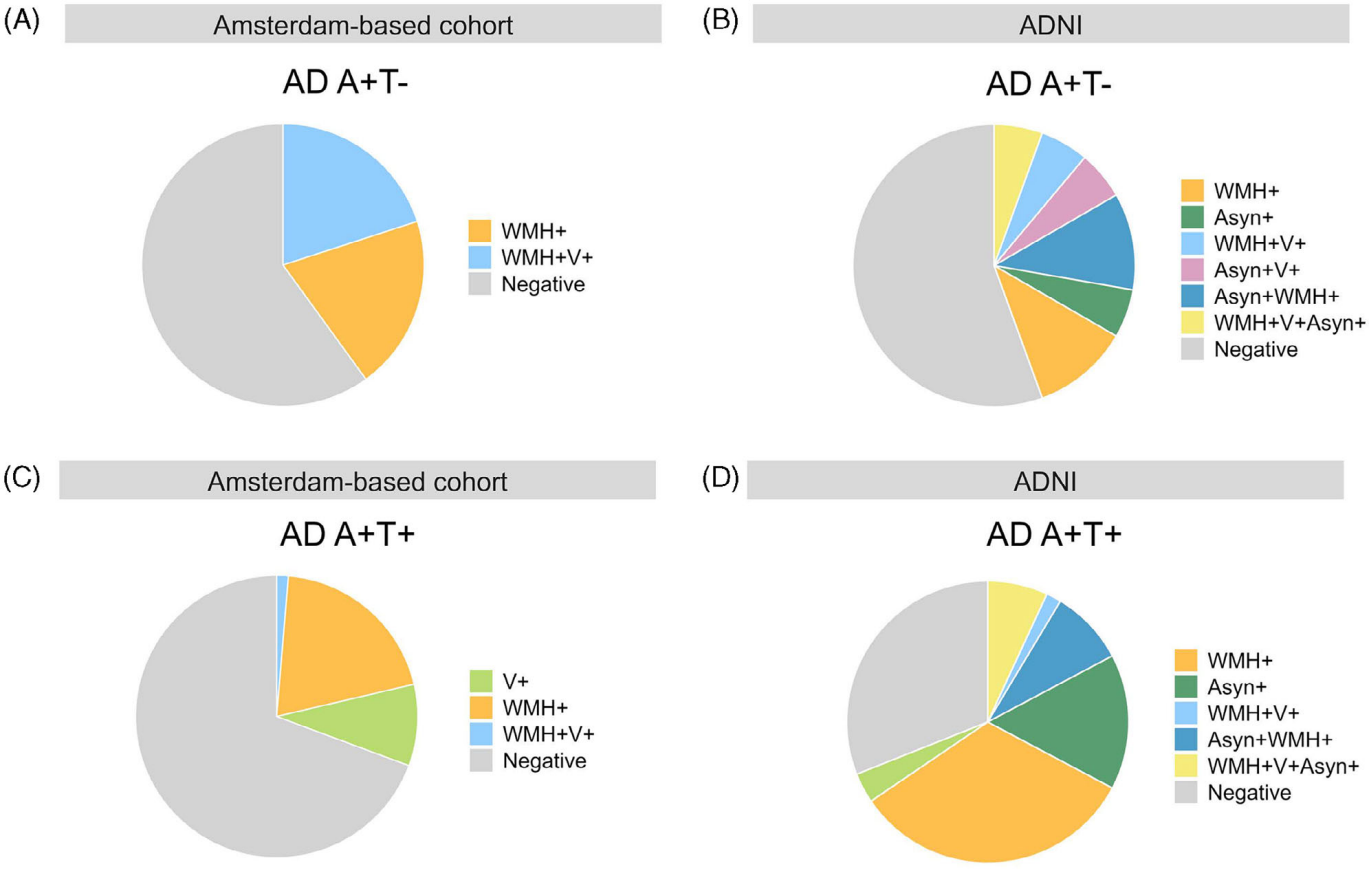
Visualizing the heterogeneity in co‐pathologies in AD A+T− and AD A+T+. (A) Pie chart showing co‐pathologies in AD A+T− in Amsterdam‐based cohort. (B) Pie chart showing co‐pathologies in AD A+T− in ADNI. (C) Pie chart showing co‐pathologies in AD A+T+ in Amsterdam‐based cohort. (D) Pie chart showing co‐pathologies in AD A+T+ in ADNI. WMH‐positivity based on >1.5 SD with reference to subset of <65‐year CU A−T−. Alpha‐synuclein status was only available in ADNI. Missing values are reported as negative. In the Amsterdam‐based cohort, WMH status was missing for one subject (10%) in AD A+T− and two subjects (4.55%) in AD A+T+. In ADNI the missing values in AD A+T− were 1 (5.56%) WMH status, 4 (22.22%) V status, and alpha‐synuclein status was missing in six subjects (33.33%). In ADNI the missing values in AD A+T+ were 13 (22.41%) for V, 1 (1.72%) for WMH, and alpha‐synuclein status was missing in 14 (24.14%) subjects. ADNI, Alzheimer's Disease Neuroimaging Initiative; Asyn, alpha‐synuclein; V, vascular/infarct status; WMH, white matter hyperintensities.

In ADNI, AD A+T− had fewer WMHs compared to AD A+T+ (*p* = 0.011) and did not significantly differ from either CU group (CU A−T−: *p* = 0.483, CU A+T−: *p* = 0.503) (Table 1, Figures S2 and S3). In the Amsterdam‐based cohort, AD A+T− had significantly more WMHs compared to CU A+T− (*p* = 0.0123) but was not significantly different from AD A+T+ (*p* = 0.329). In addition, AD A+T− had significantly more infarcts compared to CU A−T− (*p* = 0.03) but no difference compared to AD A+T+ or CU A+T−. In ADNI, there were no significant differences in the presence of infarcts. Out of the ADNI participants that had data available on alpha‐synuclein pathology, alpha‐synuclein pathology was detected in 41.7% of AD A+T− and in 40.9% of AD A+T+ but only in 15.2% of CU A−T− and in 18.3% of CU A+T+ groups (missing reported in Table S7). There was no significant difference in the prevalence of alpha‐synuclein pathology between AD A+T− and AD A+T+, indicating no more severe co‐pathology in AD A+T−. In AD A+T− alpha‐synuclein seeds were more often detected compared to CU A−T− (*p* = 0.033). Finally, we aimed to integrate our findings by visualizing the potential heterogeneity that is present within the AD A+T− group. The presence of (concomitant) co‐pathologies in AD A+T− and AD A+T+ is visualized in Figure 4. It is important to note that multiple pathologies are common in the AD A+T− group and, specifically, that the presence of pathologies is very heterogeneous across the group, underscoring the variability within AD A+T−. The exact numbers and percentages can be found in Table S7.

### 3.6 Additional analyses in ADNI: amyloid‐PET load and MCI comparison

To assess how the groups compared in amyloid load and how they compared to patients with MCI, we performed additional analyses of interest that were solely possible in ADNI.

AD A+T− showed less amyloid burden compared to AD A+T+ (β = 36.4, *p* < 0.001) and, interestingly, showed more amyloid burden compared to CU A+T− (β = −21.1, *p* < 0.001) (Figure S4). As expected, AD A+T− showed a higher amyloid burden compared to CU A−T− (β = −69.3, *p* < 0.001).

AD A+T− are older than MCI A+T− (81.8 vs 75.9, *p* = 0.004). MCI A+T− and MCI A+T+ do not differ in age (74.2 vs 75.9, *p* = 0.21) or in sex, contrary to the differences observed between AD A+T− and AD A+T+ (Table S8). MCI A+T− were more likely to be an *APOE* ε4 carrier compared to MCI A+T+ (*p* = 0.004) and had significantly more WMHs compared to AD A+T− (*p* = 0.026).

MCI A+T− showed comparable levels of cross‐sectional tau load and longitudinal tau accumulation to AD A+T− (Figure S5A,B, Table S9). However, MCI A+T− did show significantly higher hippocampal volume and better cognitive scores compared to AD A+T− (Figure S5C,D, Tables S10 and S11). Additional comparisons with other groups can be found in Tables S9, S10, and S11.

## 4 DISCUSSION

We aimed to characterize VR tau‐PET‐negative patients with amyloid‐confirmed AD dementia. In two cohorts we found that VR tau‐PET‐negative AD patients were older and had less cognitive impairment, slower cognitive decline, and less hippocampal atrophy compared to tau‐PET‐positive AD patients. Still, across both cohorts, tau‐PET‐negative AD patients showed more cognitive decline and a smaller hippocampal volume compared to both CU groups. Certain findings were only evident in one cohort. Tau‐PET‐negative AD patients tended to be more often male compared to tau‐PET‐positive AD patients in the Amsterdam‐based cohort. Tau‐PET‐negative AD patients tended to show higher tau‐PET binding in early regions (ie, MTL) compared to controls, and in late regions compared to only biomarker negative controls, while showing less amyloid burden and less tau accumulation over time than tau‐PET‐positive AD patients in the Amsterdam‐based cohort but similar levels of tau accumulation in ADNI. Tau‐PET‐negative AD patients tended to have higher global cortical thickness compared to tau‐PET‐positive AD patients in ADNI. On a group level, we did not find substantial evidence that tau‐PET‐negative AD patients showed more co‐pathology than tau‐PET‐positive AD patients. However, within the AD A+T− group, we did find substantial heterogeneity in the presence of co‐pathologies. Overall, this indicates that tau‐PET‐negative patients with a clinical AD diagnosis are on a milder disease trajectory compared to tau‐PET‐positive AD patients, but they still show evidence of cognitive decline and neurodegeneration.

Using the FDA‐approved [^18^F]flortaucipir PET VR method, ± 11% to 24% of AD patients were VR tau‐PET negative, which is consistent with previous estimates.^13^, ^14^, ^42^ A recent study found around 43% of cognitively impaired individuals to be tau‐PET negative who were older and more often male, in line with our findings.^21^ It is important to note that the majority of tau‐PET‐negative participants in the previous study had a MCI diagnosis and, thus, presumably more early‐stage tau pathology and could still develop tau pathology.^21^ By contrast, in our study we focused on the dementia cases, as we think this is a clinically relevant subgroup. When assessing the characteristics of the MCI tau‐negative group in the ADNI cohort, it was striking that they were younger than tau‐negative AD patients and similar in age to tau‐positive MCI patients (contrary to the difference between AD A+T− and AD A+T+), which indeed suggests that tau‐PET negativity in AD is different from tau‐PET negativity in MCI. The differences in prevalence may also be attributable to differences in the definition of tau positivity, as the previous study defined tau‐PET status using a global quantitative threshold, while we used the FDA‐approved VR method. Moreover, our findings align with a multicohort study showing that tau‐PET‐negative AD patients were older^13^ and previous research that found a negative correlation between tau‐PET load and age, indicating lower tau‐PET binding in older individuals.^43^, ^44^ Similarly, women were previously found to have higher tau‐PET levels compared to men.^45^

A subset of older individuals has been shown to have focal MTL tau‐PET binding,^43^, ^44^ a region that does not contribute to a positive tau‐PET VR in the FDA‐approved method. Accordingly, in our quantitative approach, we found increased levels of tau binding in early‐stage tau regions in tau‐PET‐negative AD patients in ADNI only, and minimally in late regions, indicating that these individuals tend to show higher levels of tau in early‐stage regions, which may be missed using the VR method. Accordingly, A+T−N− individuals showed higher levels of tau binding compared to A−T−N−.^46^ Over time, VR tau‐PET‐negative AD participants did not show differences in tau accumulation in the MTL or late tau regions compared to controls. This should be interpreted with caution due to the low sample size with longitudinal tau‐PET data. Furthermore, VR tau‐PET‐negative AD patients showed similar levels of tau accumulation compared to tau‐PET‐positive AD patients in early‐ and late‐stage tau regions in ADNI but showed significantly less longitudinal tau accumulation in the Amsterdam‐based cohort. Similarly, Aβ‐positive tau‐negative individuals with MCI and AD dementia showed similar levels of tau accumulation in the entorhinal cortex but lower levels of tau accumulation in medial temporal regions and a global ROI.^21^

Consistent with previous research,^21^, ^47^ we found less hippocampal atrophy in VR tau‐PET‐negative AD patients compared to tau‐PET positive ones but a lower hippocampal volume compared to both control groups, indicating an ongoing neurodegenerative process. In addition, in the current study, we analyzed longitudinal cognitive data for up to 21 years. We found less cognitive decline in both memory and non‐memory domains in tau‐PET‐negative compared to tau‐PET positive AD patients, indicating a more stable cognitive profile without an apparent profile, consistent with a large body of research finding close associations of tau with longitudinal cognition.^2^, ^4^, ^5^, ^9^, ^48^ The subtle differences in domain‐specific longitudinal decline between ADNI and the Amsterdam‐based cohort could potentially reflect cohort differences. This prognostic information could be important for patients. Previously, tau‐PET‐negative (by quantitative cut‐off) AD patients were found to have higher cross‐sectional MMSE scores,^13^ and tau‐PET‐negative MCI and AD dementia individuals were found to show less decline on MMSE.^21^ This may be partly due to the fact that the tau‐PET‐positive group consisted of 45% dementia cases, compared to 28% in the tau‐PET‐negative group, highlighting the importance of replicating these findings in AD dementia patients.^21^ These findings indicate that tau‐PET‐negative AD patients show neurodegenerative and cognitive processes consistent with the AD trajectory but are potentially earlier in the disease trajectory and have a milder disease progression.

Alternatively, since tau is closely associated with cognition, individuals with dementia with low tau may not be on the AD trajectory; instead, non‐AD co‐pathologies may contribute to their cognitive decline.^19^ Tau‐negative AD patients did not show significantly more WMHs, infarcts, or alpha‐synuclein pathology compared to tau‐PET‐positive AD patients, lowering the probability of Aβ‐positive tau‐PET‐negative dementia patients being on a non‐AD trajectory. We found ±40% of both AD dementia groups to be alpha‐synuclein positive. Previously, 4.1% of cognitively impaired inividuals were A+T− and alpha‐synuclein‐positive compared to 11.1% being alpha‐synuclein‐positive and A+T+,^17^ with other studies estimating alpha‐synuclein seeds in 30% of AD cases^49^ and *post mortem* studies, suggesting Lewy body pathology in up to 60%.^16^, ^50^ The lack of evidence for significant co‐pathology in Aβ‐positive tau‐PET‐negative compared to tau‐PET‐positive AD patients could in part be explained by the heterogeneity of co‐pathology in the group, making it difficult to detect significant differences in this minority of AD patients. Moreover, multiple pathologies are common in the AD A+T− group but are very heterogeneous across the group, underscoring the variability within AD A+T−. Still, we cannot exclude the contribution of other co‐pathologies, such as TDP‐43, of which no in vivo biomarker is available (except a temporo‐limbic fluorodeoxyglucose‐PET signature^51^). It has been suggested that A+T− individuals presenting with progressive amnestic dementia presents early ADNC with comorbid LATE‐NC (TDP‐43 pathology).^52^ However, we did not find disproportional hippocampal atrophy in tau‐PET‐negative AD patients despite their older age. Only one neuropathological case was available. *Ante mortem*, this subject showed the lowest tau level of the whole AD A+T− group. On neuropathology, the case showed Aβ pathology Thal stage 5 (A3) and tau Braak II (B2), no neuritic plaques (C0), and no TDP‐43 pathology or any other co‐pathologies, resulting in low ADNC and confirming in vivo findings.

Another explanation for cognitive impairment with relatively low tau levels could be that tau‐PET‐negative AD patients have lower cognitive or brain resilience, making them more susceptible to cognitive decline at lower levels of pathology. Previously, we found that the relationship between tau and cognition is weaker in late‐onset compared to early‐onset AD.^53^ As tau‐PET‐negative AD patients are older, lower cognitive or brain resilience or co‐pathologies may contribute to increased vulnerability.

The strengths of this study are the inclusion of two independent cohorts and their detailed characterization including a wide variety of neuroimaging and cognitive data with up to 21 years of follow‐up. Nevertheless, these cohorts are difficult to compare due to the differences in the sample, which may explain the between‐cohort discrepancies. We found a higher percentage of tau‐PET‐negative AD patients in ADNI, which may be explained by the fact that this is an older population, while the Amsterdam‐based cohort is generally younger. Second, we used the VR method instead of a quantitative threshold to define tau‐PET positivity. It is important to study the easily accessible VR method as it can be implemented widely in clinical settings. Finally, in our Amsterdam‐based cohort, we quantified tau binding with BP_ND_, allowing for a more accurate assessment of tau‐PET levels.

The study had several limitations. Its main limitation is the small sample size of the tau‐PET‐negative AD group. While some trends in tau accumulation over time were evident, the small sample size may have compromised the ability to detect certain effects. Nevertheless, this may be inherent in the group. Heterogeneity in the AD A+T− group may also limit the possibility of finding group‐level differences. Moreover, only limited longitudinal tau‐PET data were available in the Amsterdam‐based cohort. Future studies should include more participants with a longer follow‐up. Furthermore, it could be interesting to validate findings in a larger neuropathological cohort and investigate the contribution of other co‐pathologies and neuroinflammation. Finally, our cohorts had differences in tau‐PET methodology, which may explain discrepancies in findings.

In conclusion, VR tau‐PET‐negative individuals with AD dementia are older and have less cognitive decline than VR tau‐PET‐positive AD patients, demonstrating a less severe disease trajectory than tau‐PET‐positive individuals with AD dementia. On the other hand, VR tau‐PET‐negative individuals with AD dementia show more cognitive decline and atrophy compared to controls, indicating underlying neurodegenerative processes. There is no clear evidence for more co‐pathology in the tau‐PET‐negative individuals with AD dementia diagnosis, although it is possible that the presence of co‐pathologies within the group is more heterogeneous. These findings are important for patient prognosis and potential selection for future disease‐modifying therapies.

## CONFLICT OF INTEREST STATEMENT

A.B. has received research funding/support from Alzheimer Nederland, Alzheimer Association, Weston Brain Institute, Selfridges Group Foundation, Stichting Dioraphte, and Health Holland. All funding has been paid to the institutions. L.E.C. has acquired research support from GE Healthcare and Springer Healthcare (paid by Eli Lilly), both paid to the institution. L.E.C.'s salary is supported by the MSCA Postdoctoral fellowship (101108819) and Alzheimer Association Research Fellowship (23AARF‐1029663) grants. Research programs of W.M.F. were funded by ZonMW, NWO, EU‐JPND, EU‐IHI, Alzheimer Nederland, Hersenstichting CardioVascular Onderzoek Nederland, Health∼Holland, Topsector Life Sciences & Health, Stichting Dioraphte, Gieskes‐Strijbis fonds, Stichting Equilibrio, Edwin Bouw fonds, Pasman Stichting, Stichting Alzheimer & Neuropsychiatrie Foundation, Philips, Biogen MA Inc., Novartis‐NL, Life‐MI, AVID, Roche BV, Fujifilm, Eisai, and Combinostics. W.M.F. holds the Pasman chair. W.M.F. is the recipient of ABOARD, which is a public‐private partnership receiving funding from ZonMW (73305095007) and Health∼Holland, Topsector Life Sciences & Health (PPP‐allowance; #LSHM20106). W.M.F. is the recipient of TAP‐dementia (www.tap‐dementia.nl), receiving funding from ZonMw (10510032120003). TAP‐dementia receives co‐financing from Avid Radiopharmaceuticals and Amprion. All funding is paid to her institution. W.M.F. has been an invited speaker at Biogen MA Inc., Danone, Eisai, WebMD Neurology (Medscape), NovoNordisk, Springer Healthcare, and European Brain Council. W.M.F. is a consultant to Oxford Health Policy Forum CIC, Roche, Biogen MA Inc., and Eisai. W.M.F. participated in advisory boards of Biogen MA Inc., Roche, and Eli Lilly. W.M.F. is a member of the steering committee of EVOKE/EVOKE+ (NovoNordisk). All funding is paid to her institution. W.M.F. is a member of the steering committee of PAVE and Think Brain Health. W.M.F. was associate editor of Alzheimer, Research & Therapy in 2020/2021. W.M.F. is associate editor at Brain. R.O. has received research funding/support from European Research Council, ZonMw, NWO, National Institutes of Health, Alzheimer's Association, Alzheimer Nederland, Stichting Dioraphte, Cure Alzheimer's fund, Health Holland, ERA PerMed, Alzheimerfonden, Hjarnfonden, Avid Radiopharmaceuticals, Janssen Research & Development, Roche, Quanterix and Optina Diagnostics, has given lectures in symposia sponsored by GE Healthcare, is an advisory board member for Asceneuron, and a steering committee member for Bristol Myers Squibb. All the aforementioned have been paid to the institutions. R.O. is an editorial board member of Alzheimer's Research & Therapy and the *European Journal of Nuclear Medicine and Molecular Imaging*. E.G. has received research support from NWO, ZonMw, Hersenstichting, Alzheimer Nederland, Health∼Holland, and KWF. E.G. has performed contract research for Heuron Inc. and Roche. E.G. has a consultancy agreement with IXICO and Life Molecular Imaging for reading of PET scans. P.S. is an employee of EQT Life Sciences (formerly LSP). E.G.B.V. is the PI for DIAN trials, WashU, ACI, Alnylam, CogRX Therapeutics, New Amsterdam Pharma, Janssen, Roche, Vivoryon, ImmunoBrain, Alector, Biogen, BMS, Prothena, GSK, Aviadobio, Treeway. E.G.B.V. is consultant for New Amsterdam Pharma, Treeway, Vivoryon, Biogen, Vigil Neuroscience, ImmunoBrain Checkpoint, Roche, Eli Lilly en Esai. E.G.B.V. has received research support from NWO, ZonMw, Hersenstichting, and Health∼Holland. F.B. is a steering committee or Data Safety Monitoring Board member for Biogen, Merck, Eisai, and Prothena, an advisory board member for Combinostics, and Scottish Brain Sciences, and a consultant for Roche, Celltrion, Rewind Therapeutics, Merck, Bracco. He has research agreements with ADDI, Merck, Biogen, GE Healthcare, and Roche and is co‐founder and shareholder of Queen Square Analytics LTD. R.M.R., E.M.C., L.A.K., D.V., E.N., S.S.V.G., P.J.V. and R.B. have no disclosures. Author disclosures are available in the Supporting Information.

## CONSENT STATEMENT

The included studies from the Amsterdam‐based cohort were approved by the ethical review board of the Vrije Universiteit Medical Center. The ADNI protocols were approved by each cohort's respective institutional ethical review board. All participants provided written informed consent. The studies were performed in accordance with the Declaration of Helsinki and its later amendments.

## Supporting information



Supporting information

Supporting information
