# Supplementary material for: Characterizing visual read tau‐PET‐negative participants with Alzheimer's disease dementia
Source: Alzheimers Dement. 2025 Apr 12;21(4):e14423. doi: 10.1002/alz.14423 (PMC11992537; doi:10.1002/alz.14423)
Supplement: Supplementary file 1 — Supporting information [file ALZ-21-e14423-s001.docx]

**Supplementary tables and figures**

**Supplementary table 1** Baseline and longitudinal tau-PET binding

|  | | | **Amsterdam Based Cohort (BP_ND_)** | | **Amsterdam Based Cohort (SUVR)** | | **ADNI**  **(SUVR)** | |
| --- | --- | --- | --- | --- | --- | --- | --- | --- |
| **Crosssectional** | | |  |  |  | |  |  |
| ***Early tau ROI*** | | |  |  |  | |  |  |
|  | | CU A-T- | β= -0.35 | P=0.058 | β= -0.38 | **P=0.040** | β=-0.59 | **P<0.001** |
|  | | CU A+T- | β= -0.08 | P=0.664 | β= -0.10 | P=0.617 | β=-0.45 | **P=0.008** |
|  | | AD A+T+ | β=1.56 | **P<0.001** | β= 1.55 | **P<0.001** | β=1.95 | **P<0.001** |
| ***Late-stage tau ROI*** | | |  |  |  | |  |  |
|  | | CU A-T- | β= -0.30 | P=0.159 | β= -0.33 | P=0.105 | β= -0.45 | **P=0.008** |
|  | | CU A+T- | β= -0.20 | P=0.358 | β= -0.23 | P=0.274 | β=-0.29 | P=0.098 |
|  | | AD A+T+ | β=1.23 | **P<0.001** | β=1.26 | **P<0.001** | β=1.77 | **P<0.001** |
| **Longitudinal** | | |  |  |  | |  |  |
| ***Early-stage tau ROI*** | | |  |  |  | |  |  |
|  | CU A-T- | | β=0.005 | P=0.929 | β= -0.02 | P=0.805 | β= -0.04 | P=0.482 |
|  | CU A+T- | | β=0.05 | P=0.450 | Β= 0.05 | P=0.493 | Β= 0.002 | P=0.969 |
|  | AD A+T+ | | β= 0.12 | **P=0.045** | β= 0.08 | P=0.171 | β= -0.05 | P=0.576 |
| ***Late-stage tau ROI*** | | |  |  |  | |  |  |
|  | CU A-T- | | β=0.004 | P=0.932 | β=0.02 | P=0.746 | β=-0.03 | P=0.562 |
|  | CU A+T- | | β=0.03 | P=0.616 | β=0.05 | P=0.395 | β=-0.02 | P=0.793 |
|  | AD A+T+ | | β=0.14 | **P=0.007** | β=0.15 | **P=0.004** | β=0.11 | P=0.154 |

The reported estimates and p-values are derived from values of an age- and sex-adjusted linear mixed model with AD A+T- as reference group.

**Supplementary table 2** Average baseline [^18^F]flortaucipir SUVR in the Amsterdam-based cohort

|  | | **Amsterdam-based Cohort (SUVR)** |
| --- | --- | --- |
| ***Early-stage tau ROI*** | |  |
|  | CU A-T- | 1.07 (0.08) |
|  | CU A+T- | 1.15 (0.13) |
|  | AD A+T- | 1.17 (0.13) |
|  | AD A+T+ | 1.60 (0.22) |
| ***Late-stage tau ROI*** | |  |
|  | CU A-T- | 1.08 (0.06) |
|  | CU A+T- | 1.10 (0.06) |
|  | AD A+T- | 1.11 (0.10) |
|  | AD A+T+ | 1.78 (0.44) |

Values are shown as are shown as mean (SD).

**Supplementary table 3** Baseline z-scored cognitive domain scores

|  | | **Amsterdam-based Cohort** | |  |  | **ADNI** |
| --- | --- | --- | --- | --- | --- | --- |
| ***Memory*** | |  | | ***Memory*** | |  |
|  | CU A-T- | 0.28 (0.93) | |  | CU A-T- | 1.10 (0.66) |
|  | CU A+T- | -0.11 (1.12) | |  | CU A+T- | 1.0 (0.57) |
|  | AD A+T- | -1.85 (0.88) | |  | AD A+T- | -0.42 (0.64) |
|  | AD A+T+ | -2.16 (0.90) | |  | AD A+T+ | -0.94 (0.59) |
| ***Non-memory*** | |  | | ***Executive functioning*** | |  |
|  | CU A-T- | 0.01 (0.82) | |  | CU A-T- | 1.19 (0.82) |
|  | CU A+T- | -0.22 (1.06) | |  | CU A+T- | 0.90 (0.70) |
|  | AD A+T- | -2.84 (3.21) | |  | AD A+T- | 0.04 (1.27) |
|  | AD A+T+ | -3.40 (3.58) | |  | AD A+T+ | -0.92 (0.91) |
|  |  |  |  | ***Language*** | |  |
|  |  |  |  |  | CU A-T- | 0.93 (0.77) |
|  |  |  |  |  | CU A+T- | 0.85 (0.70) |
|  |  |  |  |  | AD A+T- | -0.13 (0.83) |
|  |  |  |  |  | AD A+T+ | -0.74 (1.00) |

Baseline defined as timepoint closest to PET. Values are shown as are shown as mean z-score (SD).

**Supplementary table 4** Baseline and longitudinal z-scored cognitive domain scores

|  | | **Amsterdam-based Cohort** | |  |  | **ADNI** | |
| --- | --- | --- | --- | --- | --- | --- | --- |
| **Crossectional**  ***Memory*** | |  |  |  |  |  |  |
|  |  |  |  | ***Memory*** | |  |  |
|  | CU A-T- | β= 1.14 | **P<0.001** |  | CU A-T- | β= 1.44 | **P<0.001** |
|  | CU A+T- | β= 0.87 | **P<0.001** |  | CU A+T- | β= 1.42 | **P<0.001** |
|  | AD A+T+ | β= -0.66 | **P<0.001** |  | AD A+T+ | β= -0.68 | **P<0.001** |
| ***Non-memory*** | |  |  | ***Executive functioning*** | |  |  |
|  | CU A-T- | β= 1.37 | **P<0.001** |  | CU A-T- | β= 0.63 | **P<0.001** |
|  | CU A+T- | β= 1.32 | **P<0.001** |  | CU A+T- | β= 0.85 | **P<0.001** |
|  | AD A+T+ | β= -0.38 | P=0.252 |  | AD A+T+ | β= -1.03 | **P<0.001** |
|  |  |  |  | ***Language*** |  |  |  |
|  |  |  |  |  | CU A-T- | β= 0.95 | **P<0.001** |
|  |  |  |  |  | CU A+T- | β= 0.89 | **P<0.001** |
|  |  |  |  |  | AD A+T+ | β= -0.74 | **P<0.001** |
| **Longitudinal** | |  |  |  |  |  |  |
| ***Memory*** | |  |  | ***Memory*** |  |  |  |
|  | CU A-T- | β= 0.01 | P= 0.711 |  | CU A-T- | β=0.11 | **P<0.001** |
|  | CU A+T- | β= -0.02 | P= 0.556 |  | CU A+T- | β=0.08 | **P<0.001** |
|  | AD A+T+ | β= -0.14 | **P<0.001** |  | AD A+T+ | β= -0.06 | **P<0.001** |
| ***Non-memory*** | |  |  | ***Executive functioning*** | |  |  |
|  | CU A-T- | β= 0.11 | **P=0.003** |  | CU A-T- | β=0.07 | **P<0.001** |
|  | CU A+T- | β= 0.12 | **P=0.002** |  | CU A+T- | β=0.04 | **P=0.024** |
|  | AD A+T+ | β= -0.21 | **P<0.001** |  | AD A+T+ | β= -0.1 | **P<0.001** |
|  |  |  |  | ***Language*** |  |  |  |
|  |  |  |  |  | CU A-T- | β=0.02 | P=0.187 |
|  |  |  |  |  | CU A+T- | β=0.005 | P=0.800 |
|  |  |  |  |  | AD A+T+ | β= -0.11 | **P<0.001** |

The reported estimates and p-values are derived from the baseline values of an age-, sex- and education-adjusted linear mixed model with AD A+T- as reference group.

**Supplementary table 5** Baseline and longitudinal MMSE scores

|  | | **Amsterdam-based Cohort** | |  |  | **ADNI** | |
| --- | --- | --- | --- | --- | --- | --- | --- |
| **Crossectional** | |  |  |  |  |  |  |
|  | CU A-T- | β= 0.98 | **P<0.001** |  | CU A-T- | β= 1.46 | **P<0.001** |
|  | CU A+T- | β= 0.95 | **P<0.001** |  | CU A+T- | β= 1.43 | **P<0.001** |
|  | AD A+T+ | β= -0.86 | **P<0.001** |  | AD A+T+ | β= -1.03 | **P<0.001** |
| **Longitudinal** | |  |  |  |  |  |  |
|  | CU A-T- | β= 0.07 | **P= 0.049** |  | CU A-T- | β=0.09 | **P<0.001** |
|  | CU A+T- | β= 0.06 | P= 0.118 |  | CU A+T- | β=0.08 | **P<0.001** |
|  | AD A+T+ | β= -0.27 | **P<0.001** |  | AD A+T+ | β= -0.22 | **P<0.001** |

The reported estimates and p-values are derived from the baseline values of an age-, sex- and education-adjusted linear mixed model with AD A+T- as reference group.

**Supplementary table 6** Hippocampal volume and global cortical thickness per group

|  | | **Amsterdam-based Cohort** | | | |  |  | | **ADNI** | | |
| --- | --- | --- | --- | --- | --- | --- | --- | --- | --- | --- | --- |
| ***Hippocampal volume*** | |  | |  |  | ***Hippocampal volume*** | |  | |  | |
|  | CU A-T- | β=0.05  β=0.03  β= -0.03 | **P=0.001** | | | CU A-T-  CU A+T-  AD A+T+ | | β= 0.06  β= 0.06  β= -0.03 | | | **P<0.001** |
|  | CU A+T- |  | **P=0.025** | | |  |  |  |  |  | **P<0.001** |
|  | AD A+T+ |  | **P=0.019** | | |  |  |  |  |  | **P=0.022** |
| ***Global cortical thickness*** | |  | |  |  | ***Global cortical thickness*** | |  | | | |
|  | CU A-T- | β= 0.05  β= 0.04  β= -0.07 | P=0.328 | | | CU A-T-  CU A+T-  AD A+T+ | | β= 0.13  β= 0.13  β= -0.14 | | | **P=0.002** |
|  | CU A+T- |  | P=0.481 | | |  |  |  |  |  | **P=0.002** |
|  | AD A+T+ |  | P=0.224 | | |  |  |  |  |  | **P<0.001** |

The reported estimates and p-values are derived from the of an age- and sex-adjusted linear model with AD A+T- as reference group. For hippocampal volume, also intracranial volume was added as a covariate.

**Supplementary table 7** Copathologies in AD A+T- and AD A+T+

|  |  | Amsterdam-based cohort | ADNI |
| --- | --- | --- | --- |
| AD A+T- | WMH+ | 2 (20%) | 2 (11.1%) |
|  | V+ | 0 (0%) |  |
|  | Asyn+ | - | 1 (5.6%) |
|  | Asyn+V+ | - | 1 (5.6%) |
|  | WMH+V+ | 2 (20%) | 1 (5.6%) |
|  | Asyn+WMH+ | - | 2 (11.1%) |
|  | Asyn+V+WMH+ | - | 1 (5.6%) |
|  | No evidence | 6 (60%) | 10 (55.6%) |
| *Missing* | *WMH* | 1 (10%) | 1 (5.6%) |
|  | *V* | 0 (0%) | 4 (22.2%) |
|  | *Asyn* | - | 6 (33.3%) |
| AD A+T+ | WMH+ | 15 (20%) | 19 (32.8%) |
|  | V+ | 7 (9.3%) | 2 (3.5%) |
|  | Asyn+ | - | 9 (15.5%) |
|  | Asyn+V+ | - | 0 (0%) |
|  | WMH+V+ | 1 (1.3%) | 1 (1.7%) |
|  | Asyn+WMH+ | - | 5 (8.6%) |
|  | Asyn+V+WMH+ | - | 4 (6.9%) |
|  | No evidence | 52 (69.3%) | 18 (31%) |
| *Missing* | *WMH* | 2 (4.5%) | 1 (1.72%) |
|  | *V* | 0 (0%) | 13 (22.4%) |
|  | *Asyn* | - | 14 (24.1%) |

WMH-positivity based on >1.5 SD with reference to the subset of <65 year CU A-T-. Alpha synuclein status was only available in ADNI. Missing values are reported as negative. Abbreviations: WMH = white matter hyperintensities; V = vascular/infarct status; Asyn = alpha synuclein.

| **Supplementary table 8** Participant characteristics including MCI in ADNI | | | | | | |
| --- | --- | --- | --- | --- | --- | --- |
|  | **CU A-T- (N=267)** | **CU A+T- (N=112)** | **MCI A+T- (N=52)** | **MCI A+T+ (N=74)** | **AD A+T- (N=18)** | **AD A+T+ (N=58)** |
| Age | 71.4 (7.4) **^a^** | 74.9 (7.9) | 75.9 (7.) | 74.2 (7.2) | 81.8 (6.6) **^d^** | 76.3 (9.3) |
| Sex, M (%) | 111 (41.4) **^a^** | 44 (39.3) **^b^** | 33 (63.5) | 34 (45.9) | 12 (66.7) | 31 (53.4) |
| Education in years | 16.8 (2.3) | 16.7 (2.3) | 16.4 (2.6) | 15.7 (2.4) | 15.6 (2.9) | 15.3 (2.3) **^e^** |
| APOE ε4, n carrier (%) | 56 (23.7) **^a^** | 51 (51) | 20 (45.5) | 44 (74.6) **^c^** | 7 (46.7) | 41 (75.9) **^e^** |
| MMSE | 29.1 (1.2) **^a^** | 29.1 (1.1) **^b^** | 28.3 (1.7) | 26.7 (2.3) **^c^** | 24.5 (3.7) **^d^** | 21.8 (1.2) **^e^** |
| CSF p-tau 181 | 18.7 (6.75)**^a^** | 25.4 (12.5) | 26.2 (10.9) | 38.8 (19.9) **^c^** | 27.1 (9.51) | 36.7 (12.3) **^e^** |
| Early-stage tau ROI SUVR | 1.15 (0.08)**^a^** | 1.19 (0.09) | 1.21 (0.09) | 1.63 (0.31) **^c^** | 1.28 (0.11) | 1.77 (0.40) **^e^** |
| Late-stage tau ROI SUVR | 1.51 (0.09)**^a^** | 1.54 (0.10) | 1.55 (0.09) | 1.97 (0.40) **^c^** | 1.57 (0.11) | 2.20 (0.61) **^e^** |
| Global cortical thickness (mm) | 2.72 (0.12) | 2.70 (0.15) | 2.66 (0.15) | 2.56 (0.15) **^c^** | 2.52 (0.14) | 2.40 (0.20) **^e^** |
| Hippocampal volume (mL) | 3821.44 (437.12) **^a^** | 3736.88 (381.21) **^b^** | 3593.39 (435.22) | 3304.80 (434.18) **^c^** | 2963.95 (567.28) **^d^** | 2879.01 (454.11) **^e^** |
| WMH (log) | 0.09 (1.41)**^a^** | 0.80 (1.39) **^b^** | 1.44 (1.51) | 1.04 (1.41) | 0.99 (1.53) **^d^** | 1.62 (1.03) |
| Infarcts, n (%) | 25 (11.1) | 13 (13.0) | 8 (18.6) | 7 (14.9) | 3 (21.4) | 7 (15.6) |
| Alpha synuclein, n (%) | 30 (15.2) **^a^** | 15 (18.3) | 9 (23.1) | 11 (19.3) | 5 (41.7) | 18 (40.9) |

Age, education and MMSE are shown as mean (SD). a = significantly different between MCI A+T- and CU A-T-; b = significantly different between MCI A+T- and CU A+T-; c = significantly different between MCI A+T- and MCI A+T+; d = significantly different between MCI A+T- and AD A+T-; e = significantly MCI A+T- and AD A+T+. Abbreviations: AD = Alzheimer disease; CU = cognitively unimpaired; MMSE = Mini-Mental State Examination; WMH = white matter hyperintensities. WMH availability: CU A-T-: 112/112, CU A+T- 265/267, MCI A+T- 52/52, MCI A+T+ 71/74, AD A+T- 17/18, AD A+T+ 57/58. Infarct availability: CU A-T-: 225/267, CU A+T- 100/112, MCI A+T- 43/52, MCI A+T+ 47/74, AD A+T- 14/18, AD A+T+ 45/58. APOE ε4 availability: CU A-T-: 235/267, CU A+T- 100/112, MCI A+T- 44/52, T+ 59/74, AD A+T- 15/18, AD A+T+ 54/58. Hippocampal volume availability: CU A-T-: 200/267, CU A+T- 89/112, MCI A+T- 38/52, MCI A+T+ 51/74, AD A+T- 14/18, AD A+T+ 44/58. Global cortical thickness availability: CU A-T-: 200/267, CU A+T- 82/112, MCI A+T- 29/52, MCI A+T+ 49/74, AD A+T- 10/18, AD A+T+ 39/58. Alpha synuclein availability: CU A-T-: 197/268, CU A+T- 82/112, MCI A+T- 39/52, MCI A+T+ 57/74, AD A+T- 12/18, AD A+T+ 44/58.

**Supplementary table 9** Baseline and longitudinal tau-PET binding with MCI A+T- as reference

|  | | **ADNI** | |  |  | **ADNI** | |
| --- | --- | --- | --- | --- | --- | --- | --- |
| ***Cross-sectional***  ***Early-stage ROI*** | |  |  | **Longitudinal** | |  |  |
|  |  |  |  | ***Early-stage ROI*** | |  |  |
|  | CU A-T- | β= -0.26 | **P=0.010** |  | CU A-T- | β= -0.02 | P=0.54 |
|  | CU A+T- | β= -0.14 | P=0.22 |  | CU A+T- | β= 0.02 | P=0.47 |
|  | MCI A+T+ | β= 1.39 | **P<0.001** |  | MCI A+T+ | β= 0.21 | **P<0.001** |
|  | AD A+T- | β= 0.26 | P=0.15 |  | AD A+T- | β= 0.02 | P=0.73 |
|  | AD A+T+ | β= 1.90 | **P<0.001** |  | AD A+T+ | β= -0.02 | P=0.68 |
| ***Late-stage ROI*** | |  |  | ***Late-stage ROI*** | |  |  |
|  | CU A-T- | β= -0.23 | **P=0.034** |  | CU A-T- | β= -0.004 | P=0.88 |
|  | CU A+T- | β= -0.08 | P=0.50 |  | CU A+T- | β= 0.01 | P=0.68 |
|  | MCI A+T+ | β= 1.08 | **P<0.001** |  | MCI A+T+ | β= 0.21 | **P<0.001** |
|  | AD A+T- | β= 0.19 | P=0.31 |  | AD A+T- | β= 0.03 | P=0.59 |
|  | AD A+T+ | β= 1.80 | **P<0.001** |  | AD A+T+ | β= 0.12 | **P=0.026** |

The reported estimates and p-values are derived from the baseline values of an age- and sex-adjusted linear mixed model with MCI A+T- as reference group.

**Supplementary table 10** Hippocampal volume and global cortical thickness per group with MCI AD A+T- as reference

|  | | **ADNI** | |
| --- | --- | --- | --- |
| **Hippocampal volume** | |  |  |
|  | CU A-T- | β= 0.20 | **P= 0.004** |
|  | CU A+T- | β= 0.20 | **P= 0.009** |
|  | MCI A+T+ | β= -0.03 | **P<0.001** |
|  | AD A+T- | β= -0.04 | **P<0.001** |
|  | AD A+T+ | β= -0.07 | **P<0.001** |
| **Global cortical thickness** | |  |  |
|  | CU A-T- | β= 0.04 | P=0.095 |
|  | CU A+T- | β= 0.05 | P=0.075 |
|  | MCI A+T+ | β= -0.09 | **P=0.002** |
|  | AD A+T- | β= -0.06 | P=0.24 |
|  | AD A+T+ | β= -0.21 | **P<0.001** |

**Supplementary table 11** Baseline and longitudinal z-scored cognitive domain scores with MCI AD A+T- as reference

|  | | **ADNI** | |  |  | **ADNI** | |
| --- | --- | --- | --- | --- | --- | --- | --- |
| **Crossectional**  ***Memory*** | |  |  | **Longitudinal** | |  |  |
|  |  |  |  | ***Memory*** | |  |  |
|  | CU A-T- | β= -0.68 | **P<0.001** |  | CU A-T- | β= -0.06 | **P<0.001** |
|  | CU A+T- | β= -0.67 | **P<0.001** |  | CU A+T- | β= -0.04 | **P<0.001** |
|  | MCI A+T+ | β= -0.65 | **P<0.001** |  | MCI A+T+ | β= -0.06 | **P<0.001** |
|  | AD A+T- | β= -0.75 | **P=0.001** |  | AD A+T- | β= -0.04 | **P=0.003** |
|  | AD A+T+ | β= -1.43 | **P<0.001** |  | AD A+T+ | β= -0.10 | **P<0.001** |
| ***Executive functioning*** | |  |  | ***Executive functioning*** | |  |  |
|  | CU A-T- | β= 0.66 | **P<0.001** |  | CU A-T- | β= 0.04 | **P<0.001** |
|  | CU A+T- | β= 0.45 | **P<0.001** |  | CU A+T- | β= 0.01 | P=0.30 |
|  | MCI A+T+ | β= -0.44 | **P<0.001** |  | MCI A+T+ | β= -0.05 | **P=0.001** |
|  | AD A+T- | β= -1.79 | P=0.35 |  | AD A+T- | β= -0.03 | P=0.16 |
|  | AD A+T+ | β= -1.21 | **P<0.001** |  | AD A+T+ | β= -0.12 | **P<0.001** |
| ***Language*** | |  |  | ***Language*** | |  |  |
|  | CU A-T- | β= 0.46 | **P<0.001** |  | CU A-T- | β= 0.05 | **P<0.001** |
|  | CU A+T- | β= 0.40 | **P<0.001** |  | CU A+T- | β= 0.03 | **P=0.015** |
|  | MCI A+T+ | β= -0.45 | **P<0.001** |  | MCI A+T+ | β= -0.07 | **P<0.001** |
|  | AD A+T- | β= -0.47 | **P=0.013** |  | AD A+T- | β= 0.03 | P=0.19 |
|  | AD A+T+ | β= -1.20 | **P<0.001** |  | AD A+T+ | β= -0.08 | **P<0.001** |

The reported estimates and p-values are derived from the baseline values of an age-, sex- and education-adjusted linear mixed model with MCI A+T- as reference group.

**Supplementary figures**

**Supplementary figure 1 MMSE trajectories over time**

**
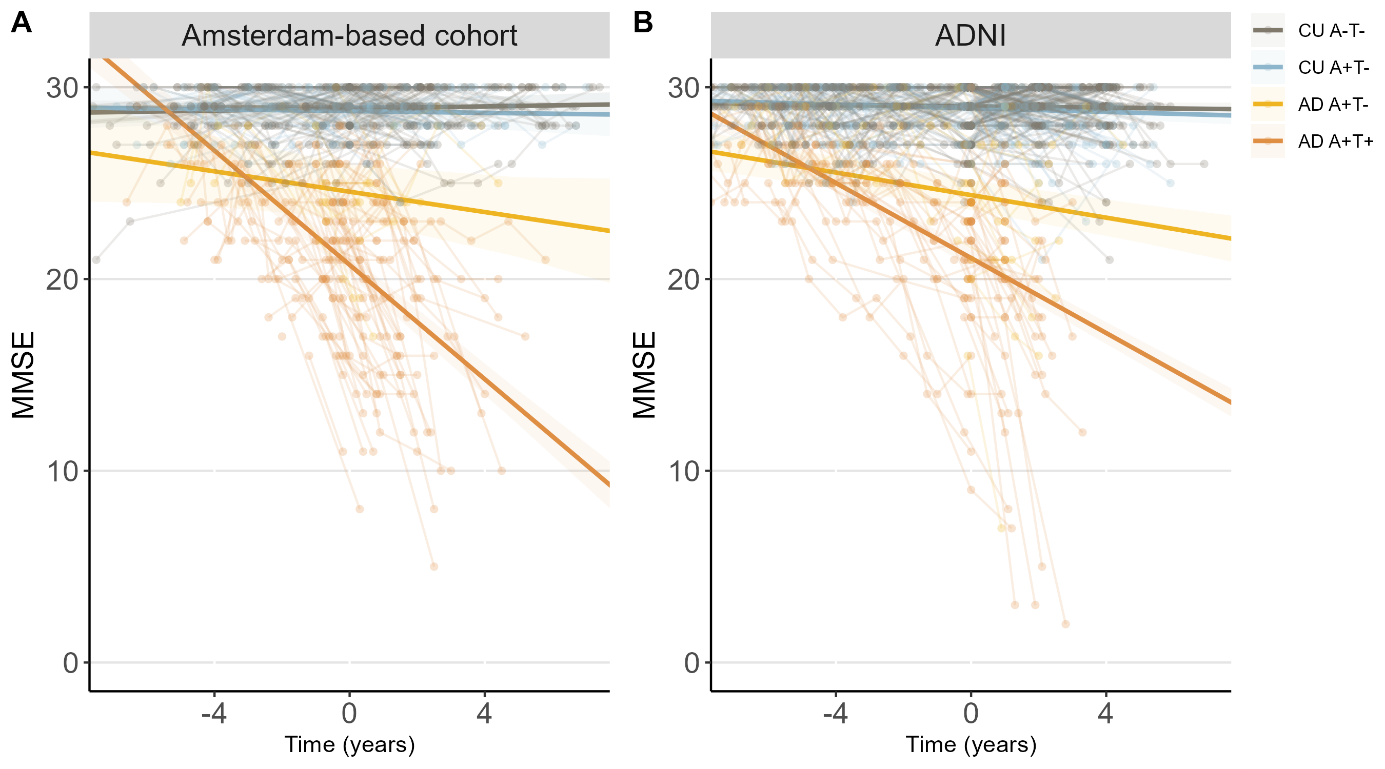
**

**Supplementary figure 2** Percentage of alpha synuclein seed positive individuals


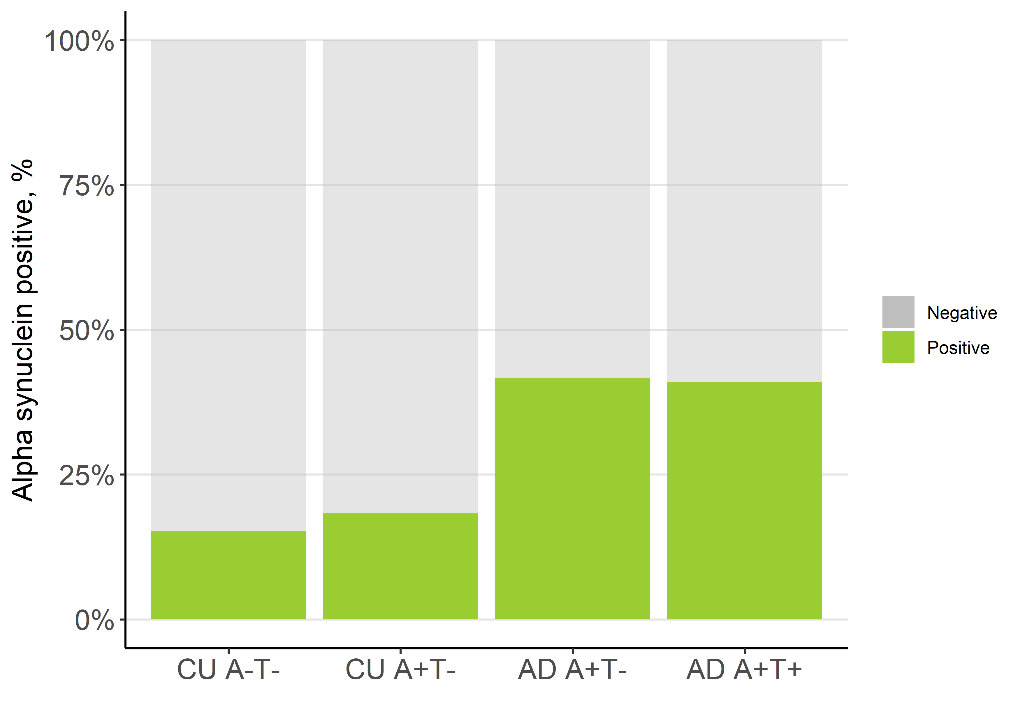


All missing values were removed for the visualization of this figure.

**Supplementary figure 3** Vascular copathology


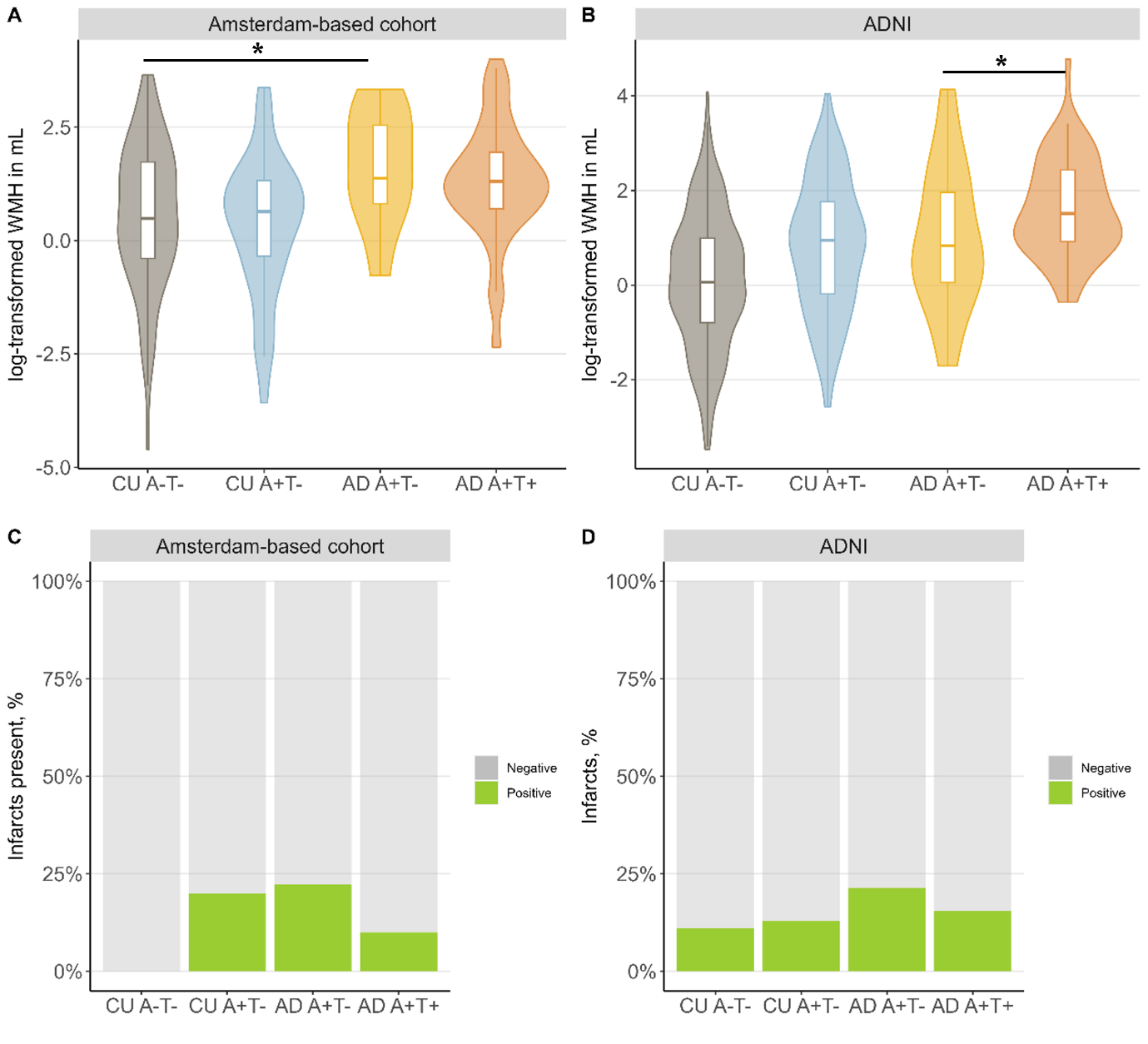


AB) Average log-transformed WMH per group. CD) Percentage of individuals with infarcts. All missing values were removed for the visualization of this figure (missing Amsterdam-based cohort 78/138, ADNI 71/456).

**Supplementary figure 4** Additional analysis in ADNI: cross-sectional centiloids by group

**
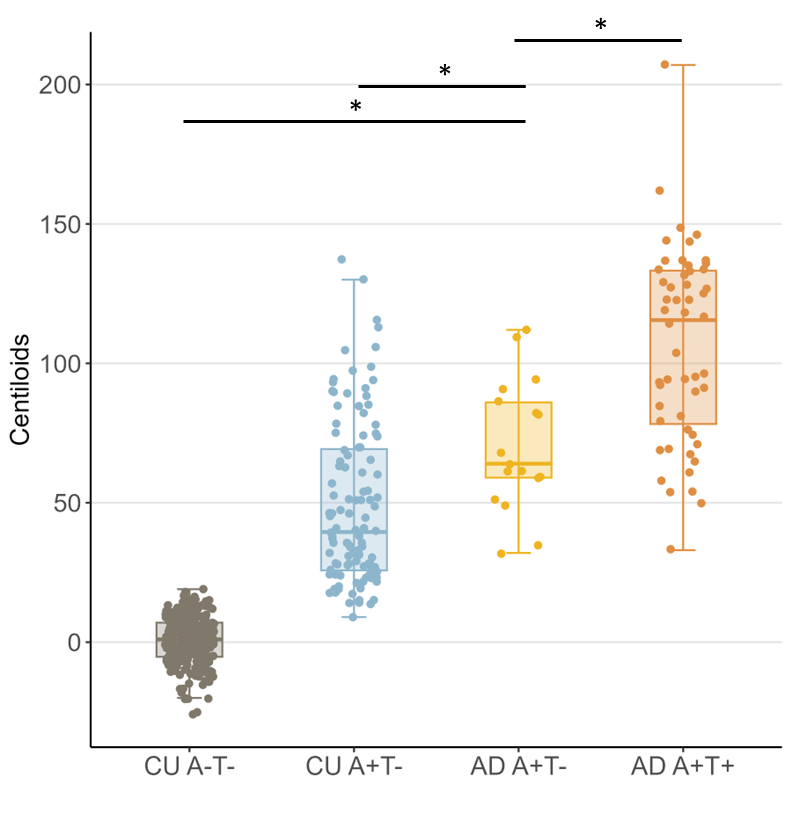
**

Boxplot showing centiloids per group. The reported p-values are derived from an age- and sex-adjusted linear model. *: p<0.05

**Supplementary figure 5** Additional analysis in ADNI: cross-sectional tau burden and atrophy in MCI

**
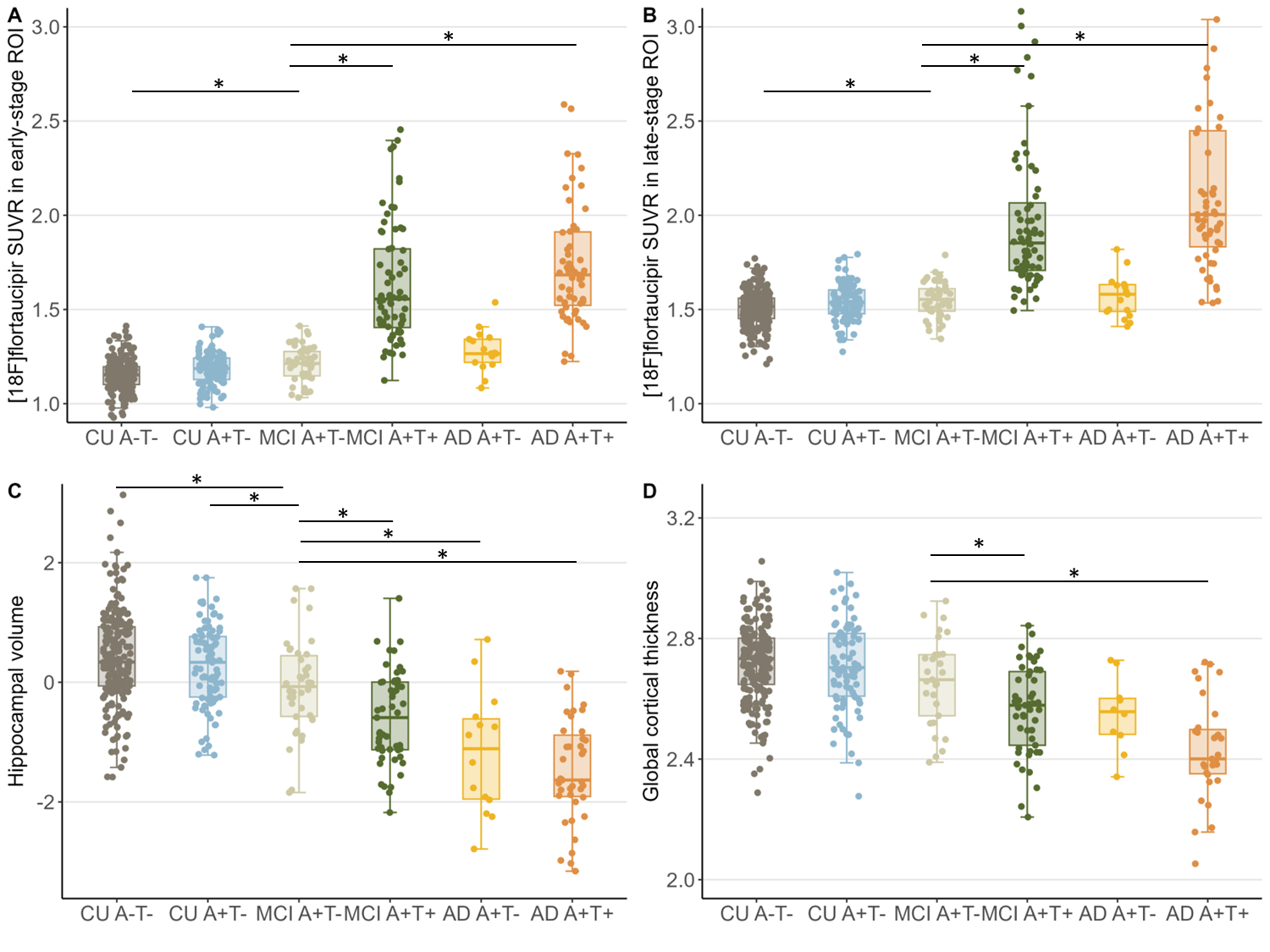
**

**A-B)** boxplots showing baseline [^18^F]flortaucipir binding in early- (MTL) and late-stage tau ROIs per group. The reported p-values are derived from the baseline values of an age- and sex-adjusted linear mixed model. **C**) Boxplot showing residuals of hippocampal volume corrected for intracranial volume. **D)** Boxplot showing global cortical thickness in mm. The reported p-values are derived from an age- and sex-adjusted linear model. *: p<0.05
